# A mixed methods exploration of self-presentation, authenticity, and role model function on Instagram: perspectives from female influencers in Germany

**DOI:** 10.3389/fpsyg.2024.1472514

**Published:** 2025-01-22

**Authors:** Daniel Zimmermann, Colleen Schneider, Kai Kaspar

**Affiliations:** Department of Psychology, University of Cologne, Cologne, Germany

**Keywords:** influencers, Instagram, self-presentation, authenticity, role model, social desirability

## Abstract

**Introduction:**

Social media influencers focussing on beauty, fashion, and fitness topics (BFFI) are important opinion leaders on Instagram. Nevertheless, they are criticized for their potential negative influence on their followers' physical and mental health due to their potentially biased self-presentation. Previous research has mainly focused on followers' perceptions of influencers' self-presentation, leaving a gap regarding the influencers' views.

**Method:**

The present survey included 26 female BFFIs from Germany (18–35 years old), with 16 self-reported micro-influencers (10,000–100,000 followers) and ten macro-influencers (>100,000 followers), representing (semi-)professionals in this domain. 17 influencers saw their main focus in the field of fitness, 16 in fashion, and 13 in beauty. The survey included demographic information, open-ended questions, established and self-developed rating scales, and a social desirability assessment to examine the BFFIs' perceptions of their goals, self-presentation, and role model function. Quantitative data was examined via inter-correlation matrix and ANOVA, and qualitative data was analyzed via an established approach for qualitative content analysis.

**Results:**

Influencers pursue mostly self-realization (50% of participants gave at least one related answer) and commercial goals (50%). While they generally perceive their self-presentation as authentic (84.6%) and positive (76.9%), they still see room for improvement in content creation (61.5%) and self-presentation (30.8%). Fear of negative feedback, absence of positivity and motivation, wrong cooperation partners (30.8% each) and insecurity (23.1%), among others, may lead to a less authentic self-presentation and use of picture editing. The tendency toward socially desirable responses (impression management) is negatively connected to the reported frequency of image editing tools used and attempts to appear authentic. Influencers reported several examples of responsible and irresponsible behaviors and strategies to avoid negative effects on their followers. The type of influencer (micro or macro level) showed a significant effect on the importance attributed to one's own role as an influencer.

**Discussion:**

The complex findings suggest potential conflicts between the influencers' goals and the expectations of followers and cooperation partners. Influencers find themselves in a dual role as users and producers. This results in concrete behavioral challenges for them, but also in implications for established theories of media use.

## 1 Introduction

Social media influencers are a new type of digital opinion leaders (Backaler, 2019). They have gained prominence and significant impact on their followers' preferences, decision-making, and behavior (Hudders et al., 2021; Morton, 2020). Instagram plays a crucial role, especially among young people. In Germany, where the present study took place, Instagram is the most frequently used social networking site among 14–29-year-olds (ARD and ZDF, 2023). Notably, influencers primarily focusing on beauty, fashion, and fitness (these three topics usually go hand in hand due to a thematic overlap) are particularly well-represented on the platform. In 2022, lifestyle profiles accounted for 14.32% of all influencers on Instagram worldwide, with 7.63% focusing on beauty and 3.16% on fitness (HypeAuditor, 2023). These influencers are perceived as role models impacting their followers' lives and value perceptions (Martínez and Olsson, 2019). So, beauty, fashion, and fitness influencers (hereinafter abbreviated to *BFFIs*) affect their followers' buying intentions of products (Nandagiri and Philip, 2018), self-presentation (Limkangvanmongkol and Abidin, 2018), and fashion choices (Casaló et al., 2020; Nurfadila, 2020). The characteristics of the influencers' self-presentation thereby are key determinants of their relationship with their followers (Leite et al., 2022) and the effectiveness of influencer marketing campaigns (Masuda et al., 2022; Guruge, 2018), underlining the importance of a well-crafted online image for influencers.

However, BFFIs have also faced criticism for their self-presentation on Instagram. Exposure to these influencers is related to increased body dissatisfaction and lower self-esteem among female (Tiggemann and Slater, 2013) and male (Tiggemann and Anderberg, 2020) consumers. While following BFFIs can be negatively connected to mental health and body image satisfaction due to constant comparisons with unreachable ideals, it can have a positive relation when perceived as inspiring and motivating (Lee et al., 2022; Panjrath and Tiwari, 2021). In principle, however, the greater focus of research to date has been on the negative effects. Consuming influencers' content is negatively connected to physical and psychological outcomes, such as body image concerns and self-objectification (Prichard et al., 2023; Pedalino and Camerini, 2022; Fardouly et al., 2018), emphasizing the need to understand BFFIs' effects on their followers and the attributes impacting these effects.

Importantly, recent research has extensively examined how followers perceive the role of influencers (e.g., Ardley et al., 2022; Tsen and Cheng, 2021; Zimmermann et al., 2022; Gómez, 2019). Young people critically perceive influencers' self-presentation and impacts on their followers, often criticizing the handling of their role model function (Zimmermann et al., 2022). In contrast, research on the influencers' perspective on their motivations, self-presentation, and roles on Instagram is relatively sparse (Kühn and Riesmeyer, 2021; Audrezet et al., 2020; Ezzat, 2020). To close this gap, the present mixed-methods study draws vital topics related to influencers from existing literature on followers' perspectives and related theories to examine the views of BFFIs themselves. For this purpose, the present study focuses on three central domains identified by previous research: goals and motives, self-presentation, and role model function of influencers.

### 1.1 Being an influencer: what goals and motives do BFFIs pursue?

The perception of influencers by their followers has played a dominant role in previous research. One focus has been on the *goals and motives* of users to follow influencers and consume their content. Specifically, the Uses and Gratification Theory (Katz et al., 1973) was applied to understand the reasons for social media use (Bawack et al., 2023; Dolan et al., 2016; Ifinedo, 2016; Whiting and Williams, 2013). This theoretical account describes how users utilize the medium that most likely will satisfy their cognitive, affective, social, and stress-release needs and motives (Katz et al., 1973). This approach ascribes an active role to the user in media selection by assuming that media users have certain psychological needs and associated motives that they try to satisfy by using mass media. This goes hand in hand with certain expectations regarding the fulfillment of these needs and the gratification of media use, which leads to a corresponding pattern in the use of media (Katz et al., 1973). Whiting and Williams (2013) identified ten uses and gratifications for utilizing social media, including social interaction, information seeking, entertainment, and knowledge about others. Regarding influencers, the motivations of users to follow them similarly include access to information about important topics, participating in discussions about the influencer's content with family and friends, being inspired by content with a positive social impact, being entertained by humorous posts, and observing the influencer's lifestyle (Morton, 2020; Kolo and Haumer, 2018). The Uses and Gratification approach has already been used to identify motives for following influencers and to show that those motives are linked to consumers (Silaban et al., 2022; Croes and Bartels, 2021) and health behavior (Alam et al., 2024). These interrelations highlight the crucial role that the gratifications that followers get from following their influencers play for the influencers themselves.

In this context, however, it is unclear what the influencer's perspective is. Previous research focused on influencers as social media users themselves and examined the reasons for users to become influencers. Reasons include a desire for fame, materialism, preference for immediate gratification over delayed gratification (Shabahang et al., 2022), financial gain, trying out new products, and enjoyment (Fetter et al., 2023). Gross and Wangenheim (2018) identified four different types of influencers with unique goals: “snoopers” who provide personal insights by creating and sharing content for self-expression, “informers” who share knowledge to educate followers about important topics, “entertainers” who provide enjoyable content for relaxation and amusement, and “infotainers” who combine information and entertainment. The motivation and goals of these influencer types determine their interaction styles with followers and their suitability for marketing campaigns (Gross and Wangenheim, 2018). What has not yet been investigated, however, are the specific goals that influencers pursue with Instagram for themselves and the goals that influencers pursue with Instagram regarding their followers. Accordingly, we asked:

***RQ1:*** What goals and motives do BFFIs pursue in relation to themselves and their followers?

### 1.2 Appearing as an influencer: how do BFFIs perceive their self-presentation?

The second crucial domain is the influencers' *self-presentation*, as they must present themselves in a certain way to appeal to their followers. Self-presentation is “the behavior that attempts to convey information about oneself or some image of oneself to other people” (Baumeister and Hutton, 1987, p. 71). The Self-Presentation Theory (Baumeister, 1982) assumes that individuals present themselves to convey information about and to build or revise an image of themselves to an audience. According to this theory, there are two main motives for self-presentation, namely self-fulfillment and getting rewards. If the audience is responsible for the potential rewards, the self-presentation is guided by the aim of ensuring that the audience perceives oneself as positively as possible (Baumeister, 1982). In the case of influencers, this audience includes their followers, potential cooperation partners, and other influencers with whom they may be competing for these cooperation partners. This can even lead to contradictory self-presentation if the demands of the different audiences differ.

The aim is to create an image of oneself that can manipulate the audience. Followers perceive influencers as credible and trustworthy sources, relying on their information about endorsed products and brands (Bello et al., 2021; Nandagiri and Philip, 2018). Young followers were shown to primarily evaluate influencers based on their credibility, appearance, and content production (Tsen and Cheng, 2021). Influencers need to be credible, close to followers (Jegham and Bouzaabia, 2022), as well as unique and original to be viewed as opinion leaders, which, in turn, can affect follower behavior toward the influencer and the advice they provide (Casaló et al., 2020). Importantly, the influencers' credibility was shown to be affected by the perceived *authenticity* of the influencers which has generally been identified as a crucial factor moderating their influence over their followers, especially in the context of influencer marketing (Shezala et al., 2024) and as a decisive characteristic by which followers evaluate influencers (Zimmermann et al., 2022; Gómez, 2019). An authentic self-presentation is thereby crucial for connecting with followers and gaining credibility (for a review, see Gómez, 2019). This can be achieved by showing consistent behavior with existing values, openness, honesty, interest in others, and acting consistently regardless of conditions or situations (Ilicic and Webster, 2016). Thus, an authentic self-presentation is a key element for building relationships between influencers and their followers and, consequently, for the success of influencer marketing campaigns.

Attractiveness and likeability have also been identified as predictors of attitude toward the influencer, with closeness to the followers moderating the relationship between likeability and attitude (Taillon et al., 2020). Balaban and Mustăţea (2019) found attractiveness to be a crucial factor in social media influencers. Given their focus on persuading followers with content emphasizing body and appearance (Pilgrim and Bohnet-Joschko, 2019), the impact of influencers' self-presentation regarding their appearance and attractiveness may be even more pronounced for BFFIs compared to influencers being active in other (business) domains, as BFFIs are the product or at least an integral part of it, highlighting the central role of their (physical) self-presentation.

The impact of influencers' self-presentation also depends on the followers' expectations. For example, inappropriate self-presentation can hurt credibility (Leite et al., 2022). Children evaluate influencers based on whether their behavior is consistent with their perceived role and self-presentation (Martínez and Olsson, 2019). This also shows the importance of aligning product endorsements with the influencer's image, as the product's image affects the influencer's image in their followers' perceptions (Balaban and Mustăţea, 2019). This makes it vital for influencers to behave in line with the expectations of followers and their social environment, that is, to present themselves in a *socially desirable* way. Indeed, Shezala et al. (2024) showed that influencers are highly socially accepted and perceived as socially desirable, underlining the importance of a socially desirable self-presentation next to authenticity.

Regarding the influencers' own perspective on self-presentation and authenticity, Gómez (2019) emphasized the need for influencers to skilfully balance their goals and self-presentation, using their self-marketing, self-promotion, and business skills to achieve social and economic capital. Influencers face the challenge of presenting themselves authentically while meeting the demands of advertisers (Hoose and Rosenbohm, 2023; Balaban and Szambolics, 2022; Arriagada and Bishop, 2021). Audrezet et al. (2020) distinguish between two strategies for dealing with brand partnerships: “passionate authenticity” where influencers are intrinsically motivated and pursue their passions and goals, and “transparent authenticity” where influencers openly discuss endorsed products and brands and disclose cooperation partners. The choice of these strategies affects the perception of authenticity by the followers (Audrezet et al., 2020). Ezzat (2020) interviewed nine social media influencers from Egypt, finding that they build unique social media profiles to attract followers and generate income, presenting themselves authentically through unique content, avoiding sensitive topics, posting pictures and selfies, and interacting with their followers while maintaining some distance.

Ethnographic research provided valuable insights in the influencer's perceptions and the importance of authenticity, stressing the intersection of commercialization and the influencer as an authentic person shaped by their mediated self-presentation (Davenport and Jones, 2021; Hund, 2019). Influencers act as brands themselves, creating identities that cater to the needs of various target audiences (Gnegy, 2017). Consequently, they present themselves in a way that maximizes marketability (Davenport and Jones, 2021), aiming to convey openness, transparency, and honesty about their personal lives, while fostering trust and a sense of community among their followers (Davenport and Jones, 2021; Gnegy, 2017). Abidin (2017) further highlights that influencers primarily attribute relatability to themselves, comprised of accessibility, believability, authenticity, and intimacy with followers. Influencers thus emphasized that they not only promote brands, but are also brands themselves and stress the importance of authentic self-presentation.

Followers, in contrast, are interested not only in the products being marketed but also in the person promoting them (Davenport and Jones, 2021). To generate trust and appear authentic, influencers must reveal aspects of their true self and life to certain extent. This authenticity plays a crucial role in their success, as it fosters the formation of relationships with followers. As a result, the influencer's persona and life experiences thus also become content consumed by followers (Davenport and Jones, 2021). Influencers are consequently not only content creators but also content themselves, a crucial factor to their success (Glatt, 2023). This also complicates the balancing between meeting the expectations of commercial partners and followers while maintaining an authentic self-presentation at the same time (Davenport and Jones, 2021). Influencers therefore report that they only promote products and brands aligned with their curated persona and their values, reflecting a sense of responsibility which they realize through thoughtful and authentic content (Davenport and Jones, 2021).

However, the influencers' challenge also seems to lie in balancing between being sufficiently authentic and avoiding overexposure, as both may evoke criticism from followers (Duffy and Hund, 2019). Influencers are aware of how their behavior and self-representation online can elicit reactions from their followers, recognizing that they expect to see not only the influencers' idealized lives, but also glimpse of their true selves and everyday realities behind the scenes (Glatt, 2023; Duffy and Hund, 2019). This dynamic can create a persistent pressure to be marketable all the time, leading the influencers to feign perfect wellbeing for fear of criticism (Glatt, 2023). When influencers deviate from their followers' expectations, they risk being perceived as inauthentic. At the same time, they face the danger that revealing too much or their genuine opinions and attitudes may provoke criticism, prompting them to self-censor (Duffy and Hund, 2019). Ultimately, influencers were found having to navigate the responsibility of deciding whether to disclose or withhold their emotions, experiences, and opinion (Davenport and Jones, 2021), a task they report being difficult (Glatt, 2023).

Thus, ethnographical grounded research has already provided valuable insights into the perception of influencers regarding their self-presentation, authenticity, and the dual role they play. We would now like to re-examine these topics using another methodological account to confirm and supplement them. Consequently, we examine how BFFIs perceive and evaluate their self-presentation in general (RQ2a), their perceived authenticity in specific (RQ2b), and if the tendency toward social desirability plays a role in this context (RQ2c). Accordingly, we asked:

***RQ2***. How do BFFIs perceive and rate their self-presentation on Instagram (RQ2a) as well as their authenticity (RQ2b), and are the influencers' perceptions connected to the tendency to respond in a socially desirable way (RQ2c)?

### 1.3 Acting as an influencer: how do BFFIs perceive their role model function?

Especially young people perceive influencers as idols and potential *role models* (Zimmermann et al., 2022; De Veirman et al., 2019). According to Social Learning Theory (Bandura, 1977), people learn new behaviors through observation. This can happen either through direct social interaction or through indirect observation, for example via media. Additionally, people perceive similar individuals as role models and are thus more likely to learn and imitate their behaviors. Furthermore, the shown outcome of the observed behavior influences the probability of imitation (Bandura, 1977). This can be even more important in the context of social media, as influencers have complete control over what they do and do not show their followers.

Influencers have a significant impact on followers' decision-making (for a review, see Hudders et al., 2021), and are perceived as persons with an impact on preferences, behavior, lifestyle, and purchase decisions (Morton, 2020). The endorsements of BFFIs can increase followers' buying intentions of certain products (Nandagiri and Philip, 2018). Previous research showed that influencers who focus on beauty topics are perceived as role models, and followers copy them (Hassan et al., 2021), especially regarding self-presentation (Limkangvanmongkol and Abidin, 2018). Similarly, influencers focussing on fashion impact the fashion choices of their followers (Casaló et al., 2020), and followers frequently base their fashion purchase decisions on the opinions of such fashion influencers, trying to imitate their style (Nurfadila, 2020). However, influencers might spread false information and demonstrate questionable and harmful behaviors, for example, hate speech, disrespect, and non-sustainable traveling habits (Hendricks and Mehlsen, 2022). Furthermore, fitness movements spread by influencers represent highly fit, slim, and lean body ideals, intending to convey a healthy lifestyle of fitness and nutrition through their pictures (Holland and Tiggemann, 2017). Though intending to motivate and inspire followers to live a healthy lifestyle, this trend also has the potential to damage the self-confidence of (young) women not corresponding to the presented fitness norm (Prichard et al., 2020; Tiggemann et al., 2018; Tiggemann and Zaccardo, 2018). Although sporty body images can motivate, they are also linked to the pathological compulsion to conform to a specific, idealized body image, which can even result in the need to change their appearance, for example, through plastic surgery (cf. RSPH, 2017). Fitness influencers may also suggest that happiness depends on a healthy, fit, and beautiful body, resulting in emulation and issues like negative self-esteem and exercise addiction (Pilgrim and Bohnet-Joschko, 2019; Raggatt et al., 2018). Young people were found to critically perceive the self-presentation of influencers and their impacts on followers, though, especially criticizing the handling of their role model function (Zimmermann et al., 2022). Instagram users were also shown to find it unlikely that influencers would abuse their power by providing false information on products (Tanha, 2020). This shows the strength and importance of influencers in general and BFFIs in specific as role models for their followers, both in a positive and a negative way.

In the context of the influencers' perspective on their role model function, Kühn and Riesmeyer (2021) conducted interviews with fitness and fashion influencers on commercialization and responsibility, finding that influencers see themselves as brand ambassadors and opinion leaders. They use social media to promote cooperation partners and brands to their followers. They also perceive themselves as role models with a responsibility toward their followers. Consequently, influencers reflect on their content based on their followers' feedback to maintain their self-perception and media persona (Kühn and Riesmeyer, 2021). Furthermore, influencers were shown to be aware of the responsibility that comes with the influence of their status (Davenport and Jones, 2021). Despite these findings, it is still not clear how BFFIs attempt to implement these roles. Thus, we asked:

***RQ3:*** How do BFFIs perceive their function as role models on Instagram, and how do they try to realize this?

**Figure 1:**
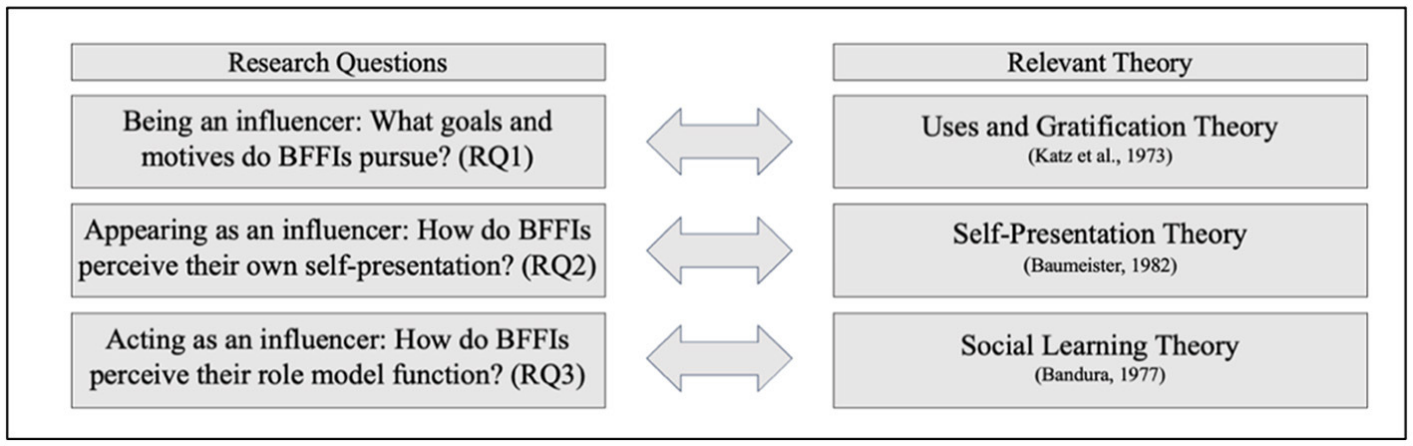
Summary of the research questions and the related theory.

Figure 1 summarizes the research questions of the present study and the corresponding theoretical accounts.

## 2 Methods

### 2.1 Participants

We conducted a mixed-methods online survey utilizing closed and open-ended questions to collect quantitative and qualitative data via the software Unipark (Tivian, 2017), which ran from June 22, 2021 to August 22, 2021. Influencers in the field of beauty, fitness, and fashion were identified via inspection of Instagram accounts and systematically contacted via direct messages on Instagram with an invitation to participate in the online survey. We deliberately contacted only female influencers who are active in this field. In fact, at the time the present study was designed and conducted, seven out of the ten biggest beauty and fashion influencers in Germany were female (Criteo, 2020). We realized that the target group is fundamentally difficult to reach and recruit for such a study. In the present study, only 34 out of 137 persons who clicked on the link to the survey completed it (24.8%). Thereby, nearly half of the dropouts canceled the survey at the first page where they were greeted and were provided information about the specific topic and aim of the survey. Inclusion criteria for participation included a minimum age of 18 and maximum age of 35 years, engaging in at least one of the topics of beauty, fitness, and fashion, self-identifying as female influencers who regularly and actively present themselves on Instagram in Germany, understanding the German language, and providing informed consent. All procedures performed in the study followed the ethical guidelines of the German Psychological Society (DGPs).

Participants were informed that their data in this study would solely be used for research purposes and that all data would be collected anonymously without any identifying information. Participation was entirely voluntary. Participants were free to end the questionnaire at any time without giving reasons. Data of participants who terminated the questionnaire prematurely were not included in the analyses. We provided contact information for inquiries of any kind. Informed consent was given by checking a box before the start of the survey.

Thirty-four participants completed the online survey, but eight of them stated that they did not identify themselves as influencers and did not appear regularly and actively on Instagram in this role. Consequently, 26 German female influencers met the inclusion criteria and thus were included in the analyses. 17 of these 26 influencers reported being active in the topic of fitness (65.4%), 16 in fashion (61.5%), and 13 in beauty (50.0%), whereby multiple answers were possible. Based on the classification of Gómez (2019), 16 participants were micro-influencers (10,000–100,000 followers)—eleven influencers with 10,000 to 50,000 followers (42.3%) and five influencers with 50,000–100,000 followers (19.2%)—and ten influencers reported being at least macro-influencers with more than 100,000 followers (38.5%). On average, the influencers had been active on Instagram for 3.5 years (*SD* = 1.8) and reported spending *M* = 227 min (*SD* = 120) per day on Instagram.

### 2.2 Materials and measures

The survey included qualitative open-ended and quantitative closed-ended questions. Unless otherwise noted, closed-ended questions asked participants to indicate their level of agreement with statements referring to various aspects of their self-presentation on Instagram on a five-point scale ranging from 1 (“don't agree at all”) to 5 (“agree very much”). All closed-ended items and their assignment to the individual research questions can be found in Table 1. Open-ended questions asked participants to provide up to five statements about specific aspects of their self-presentation on Instagram, with a limit of 150 characters per response. The questionnaire translated into English can be found in Supplementary Figure 1.

**Table 1:** All quantitative parts of the survey including mean values.

	**M (SD)**
**BFFIS' goals and motives (RQ1)**	
*Importance of being an influencer* (Cronbach's α = 0.780)	3.58 (0.87)
Being an influencer takes great importance in my life	3.69 (1.01)
The commercial factor of Instagram is important for me	3.46 (0.91)
**BFFIs' perception of their self-presentation, authenticity, and tendency to respond in a socially desirable way (RQ2a–RQ2c)**	
*Celebrity Brand Authenticity Scale* (Cronbach's α = 0.685)	4.26 (0.65)
I try to behave on Instagram in a manner that is consistent with my held values, even if others criticize or reject me for doing so	4.31 (0.88)
As an influencer on Instagram, I care about openness and honesty	4.62 (0.75)
As an influencer on Instagram, I place a good deal of importance on others understanding who I really am	3.85 (1.12)
My followers can count on me being who I am regardless of the situation	4.27 (0.83)
*Attempt to appear authentic on Instagram* (Cronbach's α = 0.701)	2.91 (0.93)
It does matter to me how I appear to my followers and how they perceive me	3.92 (1.06)
I am very authentic on Instagram and can simply be myself (negatively poled)	3.92 (1.02)
As an influencer, I sometimes just try to “appear authentic”	2.73 (1.40)
*Frequency of using picture editing tools*	
How often do you use photo editors, face filters, and other similar tools for your stories and posts on Instagram?	3.38 (1.24)
*Compatibility of picture editing software with self-presentation* (Cronbach's α = 0.895)	2.79 (1.19)
The use of face filters and beauty retouching is compatible with my intended goals of my self-presentation on Instagram	2.81 (1.23)
The use of face filters and beauty retouching is compatible with authentic self-presentation	2.77 (1.28)
*Social desirability: self-deceptive enhancement* (Cronbach's α = 0.444)	16.12 (2.83)
My first impression of people usually turns out to be correct	5.00 (1.39)
I am often unsure of my judgment (negatively poled)	5.38 (1.58)
I always know exactly why I like something	5.73 (1.12)
*Social desirability: impression management* (Cronbach's α = 0.804)	15.12 (4.90)
I've gotten too much change back before and didn't say anything (negatively poled)	4.50 (2.58)
I am always honest with others	5.08 (1.47)
I have occasionally taken advantage of someone (negatively poled)	5.54 (1.53)
**BFFIs' perception of their role model function (RQ3)**	
*Perceived role model function of influencers in general* (Cronbach's α = 0.896)	3.81 (1.00)
Influencers generally have a role model function for their followers on Instagram	4.04 (1.04)
Influencers are important as role models for their followers	3.58 (1.07)
*Perception of one's own role model function and associated responsibility* (Cronbach's α = 0.824)	4.08 (0.64)
I am aware of a certain responsibility toward my followers on Instagram	4.31 (0.79)
I am aware of my influence as an influencer on my followers	4.31 (0.79)
I know that I may also negatively influence my followers through falsely conveying ideals of my self-presentation on Instagram	4.35 (0.69)
In my opinion, my followers strongly orientate to me and my posts	3.38 (1.02)
About my self-presentation as an influencer, I demonstrate responsible handling of my role model function toward my followers	4.04 (0.87)

Most rating scales ranged from 1 to 5 and items were aggregated to form a scale by calculating mean values (across items). The two social desirability dimensions ranged from 1 to 7, negatively poled items were recoded, so higher values indicate higher self-deceptive enhancement and impression management. Following Paulhus (1984), we summed up the ratings of the three items of each social desirability dimension. 18 is the lowest cut-off point that indicates socially desirable response tendencies. None of the scales showed signs of a floor or ceiling effect.

#### 2.2.1 Demographics

Participants reported about their Instagram usage, including whether they identify as influencers who actively and regularly present themselves on Instagram (single choice: “yes/no”). Participants were asked about their average daily Instagram usage time in minutes and the number of years they have been active as influencers on Instagram (open-ended response format). Participants reported their follower number by indicating whether they are in the range of micro-influencers (two sub-groups: 10,000–50,0000 and 50,001–100,000 followers), or at the level of macro-influencers (>100,000 followers) (Gómez, 2019), as well as the content they cover on Instagram (multiple choice: “beauty”; “fitness”; “fashion”; “family”; “animals”; “food”; “travel”; “interiors”; “others”). In contrast to some of the few previous studies that have surveyed influencers (e.g., Audrezet et al., 2020; Kühn and Riesmeyer, 2021), we specifically did not invite any person who was in the range of nano-influencers (<10,000 followers) at the time of the study to have a sample of (at least) semi-professionals.

#### 2.2.2 The goals and motives that BFFIs pursue in relation to themselves and their followers (RQ1)

Participants first rated their agreement with two statements about the *importance of being an influencer* (“Being an influencer takes great importance in my life”, “The commercial factor of Instagram is important for me”, *M* = 3.58, *SD* = 0.87, Cronbach's α = 0.780). Higher values on this scale indicate a greater importance of the role as a social media influencer. We additionally asked them in an open-ended format to list up to five goals that they pursue in relation to themselves and to rank these goals in order of importance (the first goal as the most important). Finally, we asked the influencers to list up to five goals that they pursue specifically in relation to their followers and to rank these goals in order of importance.

#### 2.2.3 BFFI's perception of their self-presentation on instagram (RQ2a)

We examined how BFFIs perceive their self-presentation on Instagram using an open-ended format in three steps:

First, participants were asked to list up to five attributes (characteristics, features, traits) that best describe how they present themselves on Instagram and to rank these attributes in order of importance (the first attribute as the most important).

Second, participants were asked to take the perspective of their followers and assess which attributes related to self-presentation their followers would most likely ascribe to them. Once again, these attributes should be ordered according to importance (the first attribute is the most important).

Third, participants were asked to name up to five aspects in which they personally still see the potential for improvement in their self-presentation on Instagram and to rank these aspects in order of importance (the most relevant weakness first).

#### 2.2.4 BFFI's perception of their authenticity (RQ2b)

We investigated the BFFIs' perception of their authenticity by adapting four items from Ilicic and Webster's (2016) *Celebrity Brand Authenticity Scale* (*M* = 4.26, *SD* = 0.65, α = 0.685) for the context of influencers on Instagram (e.g., “My followers can count on me being who I am regardless of the situation”, “As an influencer on Instagram, I care about openness and honesty”). Ilicic and Webster (2016) defined that “celebrity brand authenticity […] a construct that represents consumer perceptions of celebrities being ‘true to oneself' in their behaviors and interactions with consumers” (p. 410). All items can be found in Table 1. Higher values on this scale indicate that influencers perceive themselves as an authentic brand.

To capture a different facet of authenticity, namely the deliberate or conscious *attempt to appear authentic on Instagram*, we added a scale with three self-developed items (e.g., “As an influencer, I sometimes just try to ‘appear authentic”', *M* = 2.91, *SD* = 0.93, α = 0.701). Higher values on this scale indicate a stronger attempt to appear authentic to the followers. All items are presented in Table 1.

Further, the participants indicated the *frequency of using picture editing tools* (“How often do you use photo editors, face filters, and other similar tools for your stories and posts on Instagram”) on a five-point scale ranging from 1 (“not at all”) to 5 (“always”) (*M* = 3.38, *SD* = 1.24). Higher values on this item indicate a higher frequency. In the case of participants using photo editors, they were asked to list up to five reasons for using photo editors, face filters, and other similar tools for their posts on Instagram (open-ended format).

Participants also indicated how much they agree with two statements about the *compatibility of picture editing software with self-presentation* (“The use of face filters and beauty retouching is compatible with my intended goals of my self-presentation on Instagram”; “The use of face filters and beauty retouching is compatible with authentic self-presentation”, *M* = 2.79, *SD* = 1.19, α = 0.895), see Table 1. Higher values on this scale indicate a higher perceived fit between picture editing software and the intended self-presentation as authentic.

Finally, we asked the participants to give up to five reasons why they sometimes present themselves less authentically or less “as themselves” to their followers (open-ended format).

#### 2.2.5 BFFIs' tendency to respond in a socially desirable way (RQ2c)

We assessed the participants' tendency for socially desirable answering behavior with the Balanced Inventory of Desirable Responding (BIDR) by Paulhus (1984) in the short form by Winkler et al. (2006). This instrument covers two dimensions, namely *self-deceptive enhancement* (i.e., the unconscious tendency to optimistically distort reality perception to protect the self-image, α = 0.444) and *impression management* (i.e., the conscious presentation of the best possible image of oneself to an audience, α = 0.804). Each dimension consists of three items rated on a seven-point scale ranging from 1 (“strongly disagree”) to 7 (“strongly agree”). Based on Paulhus (1984), we added up the three items of the self-deceptive enhancement scale (*M* = 16.12, *SD* = 2.83) and the impression management scale (*M* = 15.12, *SD* = 4.90), respectively, and used these sums in further analyses. Negatively poled items were recoded, so higher values on these scales indicate higher self-deceptive enhancement or impression management, respectively.

#### 2.2.6 BFFI's perception of their role model function (RQ3)

Participants responded to two closed-ended and self-developed items about their *perceived role model function of influencers in general* (“Influencers generally have a role model function for their followers on Instagram”, “Influencers are important as role models for their followers”, *M* = 3.81, *SD* = 1.00, α = 0.896), whereby higher (mean) values on this scale indicate greater recognition of the role model function of influencers.

Participants also responded to a scale including five items about their *perception of one's own role model function and associated responsibility* toward their followers on Instagram (e.g., “I am aware of a certain responsibility toward my followers on Instagram”, *M* = 4.08, *SD* = 0.64, α = 0.824, see Table 1 for all item wordings). Higher values on this scale indicate a higher awareness of their own influence on followers.

We asked participants to list up to five aspects that they felt constituted responsible behavior as an influencer on Instagram and up to five aspects of irresponsible behavior as an influencer on Instagram in order of importance (with the first aspect being the most important).

Finally, we wanted to explore the personal handling of the role function in more detail and asked the participants three questions in an open-ended format: First, they were asked to list up to five attributes (characteristics, features, traits) that best describe how they fulfill their responsibility or role model function as an influencer. Second, they were asked to name up to five aspects in which they see potentially negative influences of their self-presentation on their followers. Third, they were asked to name up to five aspects that they implement to prevent possible negative influences of their self-presentation on their followers.

### 2.3 Procedure

Before starting the actual survey, the participants were welcomed and informed about the topic and the procedure of the survey. To achieve the highest possible level of truthful information, the influencers were assured that the survey was constructed in a manner that did not require any personal identifying information. We also told participants not to provide any information that might disclose their identity, such as their Instagram account name, to ensure anonymity. After giving informed consent, the survey started.

First, participants provided information about their Instagram usage, demographics, and importance of being an influencer and of commercialization. This was followed by information on their tendency to respond in a socially desirable way, their goals and motives, as well as their perceptions on their self-presentation on Instagram, their authenticity, and their role model function, in that order. The participants were then dismissed.

### 2.4 Data analysis

Analyses were performed with SPSS 27. For quantitative data, we first conducted exploratory factor analysis (varimax rotation) for all self-developed scales (i.e., a minimum of two items) to ensure factorial validity (RQ1, RQ2a, RQ2b, RQ3). Factor loadings for the self-developed items are shown in Supplementary Table 1. The factor analyses supported single-factorial structures for all self-developed scales, with all Kaiser-Meyer-Olkin measures of sampling adequacy > 0.50 and all Bartlett's tests of Sphericity being significant (*p* ≤ 0.001). All scales had eigenvalues ≥ 1. Additionally, we found good reliability in terms of internal consistency for all self-developed scales (all α ≥ 0.701, see Table 1). In view of the low internal consistency of the self-deceptive enhancement scale (α = 0.444) of the short form of the BIDR (Paulhus, 1984) by Winkler et al. (2006), we investigated whether the removal of one item would improve reliability. However, this was not the case, so all three items were retained. As this scale had been previously validated (Winkler et al., 2006), we decided to use the scale despite its modest Cronbach's alpha. For each scale, we calculated mean values to check for potential floor or ceiling effects (RQ1, RQ2a, RQ2b, RQ2c, RQ3) and we calculated an inter-correlation matrix (RQ2c, exploratory analyses). Potential effects of influencer type in terms of number of followers were tested via ANOVA (exploratory analyses).

To analyse the qualitative data, we performed a qualitative content analysis following the standard approach by Mayring (2015), as already employed in previous studies (Hoss et al., 2021; Kaspar et al., 2014; Meier et al., 2023; Trixa and Kaspar, 2024) (RQ1, RQ2a, RQ2b, RQ3). The coding system was developed inductively and iteratively based on the responses of the first 10% of the participants. First, we derived initial higher-level categories and developed a coding manual describing the categories and the criteria for the classification into these categories by using MS Excel and MS Word. Second, the responses of the first 10% of participants were independently coded by two persons (first and second author) according to the coding system after a short introduction to ensure the coding system's applicability and objectivity. Subsequently, inter-coder reliability was assessed using Cohen's (1960) Kappa to evaluate the clarity and quality of the coding system, to identify possible sources of error, and to optimize the category system when needed. After revising the coding manual, all data were coded, and inter-coder reliability was re-assessed. We achieved very good agreement with κ > 0.89 (cf. Landis and Koch, 1977). In the few cases of disagreement, a consensual agreement was reached through discussion to allow unequivocal frequency analyses. The categories and their definition can be found in the results section. The Supplementary Tables 2A–C shows sample answers for each category.

## 3 Results

### 3.1 Being an influencer: what goals and motives do BFFIs pursue? (RQ1)

We examined the goals and motives that the BFFIs pursue by qualitative content analysis of the answers the participants provided for the open-ended questions. Table 2 shows the number of responses given by the entire sample of influencers that fell into the respective category (and percentage frequency in relation to all responses across all categories), as well as the total number of influencers who gave at least one answer of the corresponding category (and the percentage frequency in relation to the sample size, *n* = 26). In the following sections, we refer to the percentage frequency of influencers who gave at least one answer to the corresponding category, as this value represents the value adjusted for multiple answers of individual participants.

**Table 2:** Categories of influencers' goals and motives they pursue for themselves and in relation to their followers with total numbers and percentage frequencies.

**BFFIs' goals and motives (RQ1)**			
**Topic and associated response categories**	**Total**		**Short definition of category's content**
	***n**_*responses*_* **(%)**	***n**_*participants*_* **(%)**	**All answers that refer to…**
**Goals and motives that influencers pursue for themselves**			
Self-realization and self-development	22 (20.4)	13 (50)	…the positive development and self-realization.
Self-promotion and increasing public awareness level	18 (16.7)	11 (42.3)	…the marketing of oneself, a brand, and products.
Serving and acting as a role model and motivating, inspiring, and empowering followers	14 (13.0)	11 (42.3)	…serving and acting as a role model and motivating, inspiring and empowering followers.
Making money	13 (12.0)	13 (50)	…earning money, being an influencer as source of income, and finances.
Being inspired, motivated, informed, and entertained	12 (11.1)	8 (30.8)	…the inspiration, motivation, entertainment and getting information.
Establishing contact to and bidirectional exchange with others	8 (7.4)	7 (26.9)	…the bidirectional exchange and establishing contact with others.
Spreading joy and fun	5 (4.6)	4 (15.4)	…a positive influence on enhancing the fun and enjoyment for others.
Presenting educational and informative content	4 (3.7)	4 (15.4)	…the creation of high-quality content and thus the presentation of educational and informative content.
Other non-specific or rare answers	12 (11.1)	10 (38.5)	…unspecific or rare occurrences and therefore cannot be assigned to any other category.
**Goals and motives that influencers pursue in relation to their followers**			
Serving and acting as a role model and motivating, inspiring, and empowering followers	31 (30.1)	16 (61.5)	…serving and acting as a role model and motivating, inspiring and empowering followers.
Establishing contact and bidirectional exchange with others	18 (17.5)	12 (46.2)	…the bidirectional exchange and establishing contact with others.
Self-promotion and increasing public awareness level	13 (12.6)	10 (38.5)	…the marketing of oneself, a brand, and products.
Presenting educational and informative content	13 (12.6)	10 (38.5)	…the creation of high-quality content and thus the presentation of educational and informative content.
Being perceived as sympathetic, authentic, and honest	8 (7.8)	6 (23.1)	…a sympathetic and authentic perception.
Getting appreciation and acknowledgment	7 (6.8)	6 (23.1)	…to receiving appreciation and recognition.
Communicating an own point of view on a topic	5 (4.9)	4 (15.4)	…communicating one's own point of view on a topic.
Self-realization and development	4 (3.9)	3 (11.5)	…self-realization and further development.
Other non-specific or rare answers	4 (3.9)	3 (11.5)	…unspecific or rare occurrences and therefore cannot be assigned to any other category.

*n*_*responses*_ represents the number of responses given by the entire sample of influencers that fell into the respective category and (in brackets) the percentage frequency in relation to all responses across all categories. *n*_*participants*_ (%) represents the total number of influencers who gave at least one answer of the corresponding category (i.e., this value represents the number adjusted for multiple answers), as well as the percentage frequency in relation to the sample size (*n* = 26). Possible missing percentages of 100 % are due to missing answers, as participants were not forced to provide responses.

Regarding the goals and motives that BFFIs pursue for themselves on Instagram, most influencers try to achieve both, intrinsic goals and motives like self-realization and self-development (50% of participants gave at least one answer to this category) and acting as role models that motivate, inspire, and empower its followers (42.3%), and extrinsic goals and motives like making money (50%) and self-promotion by increasing public awareness level (42.3%).

Similarly, when asked about the goals and motives the influencers want to achieve in relation to their followers, they reported they mostly try to act as a role model to motivate, inspire, and empower their followers (61.5%), to establish contact and bidirectional exchange with them (46.2%), and to present educational and informative content to them (38.5%). However, they also want to promote themselves via their followers and to raise their awareness level among them (38.5%). Overall, the result pattern shows the dichotomy between intrinsic goals and motives the influencers pursue like self-realization, as well as extrinsic and commercial ones like making money and self-promotion.

We also asked the influencers to rank their answers in order of importance to them. We analyzed how often a goal/motive of a certain category was ranked first (most important), second, third, fourth and fifth by the influencers. The full results of this analysis are presented in Supplementary Table 3A. In a nutshell, self-realization (23.1% of the whole sample ranked this category as first goal) and making money (19.2%) were considered as the most important goals and motives that BFFIs pursue for themselves. In relation to their followers, most of the influencers especially see acting as a role model (42.3%), presenting educational and informative content (19.2%), and self-promotion and increasing of public awareness level (19.2%) as the most important goals and motives.

Overall, it becomes clear that influencers mainly pursue two types of goals and motives for themselves, namely self-realization and self-development on the one hand, but also economic goals such as making money on the other hand. Moreover, the influencers try to achieve similar objectives in relation to their followers. They try being a role model and empower their followers, although at the same time they utilize them for self-promotion and to raise public awareness.

### 3.2 Appearing as an influencer: how do BFFIs perceive their self-presentation? (RQ2)

We investigated how the BFFIs perceive and rate their self-presentation (RQ2a), the authenticity of their self-presentation (RQ2b), and if the tendency to answer in a socially desirable way is connected to that (RQ2c).

#### 3.2.1 BFFIs' perception of their self-presentation (RQ2a)

Table 3 shows the results of the qualitative content analysis of open-ended questions regarding the perception of self-presentation. First, the influencers were asked to list up to five attributes that best describe how they present themselves on Instagram. Most of the influencers described their self-presentation as authentic, real, and approachable (84.6% of participants gave at least one answer to this category), entertaining, positive, and motivating (76.9%), confident (50.0%), and physically attractive, stylish, and sporty (30.8%).

**Table 3:** Categories of influencers' perceived self-presentation with total numbers and percentage frequencies.

**BFFIs' perceived self-presentation (RQ2a, RQ2b)**			
**Topic and associated response categories**	**Total**		**Short definition of category's content**
	***n**_*responses*_* **(%)**	***n**_*participants*_* **(%)**	**All answers that refer to…**
**Influencers' description of own self-presentation**			
Authentic, real, and approachable	34 (30.9)	22 (84.6)	…an authentic, real, and approachable self-presentation.
Entertaining, positive, and motivating	29 (26.4)	20 (76.9)	…an emotionally positive communication and motivating self-presentation.
Confident	16 (14.5)	13 (50.0)	…a self-confident self-presentation.
Physically attractive, stylish, and sporty	11 (10.0)	8 (30.8)	…appearance and the physical self-presentation.
Ambitious	7 (6.4)	7 (26.9)	…an ambitious self-presentation.
Perfectionistic	3 (2.7)	3 (11.5)	…perfectionist characteristics, traits, and attributes of the self-presentation.
Other non-specific or rare answers	10 (9.1)	6 (23.1)	…unspecific or rare occurrences and therefore cannot be assigned to any other category.
**Influencers' beliefs of followers' perception of own self-presentation**			
Entertaining, positive, and motivating	30 (29.1)	18 (69.2)	…an emotionally positive communication and motivating self-presentation.
Authentic, real, and approachable	28 (27.2)	17 (65.4)	…an authentic, real, and approachable self-presentation.
Arrogant and narcissistic	14 (13.6)	11 (42.3)	…arrogance and negative emotional communication.
Physically attractive, stylish, and sporty	10 (9.7)	7 (26.9)	…appearance and the physical self-presentation.
Confident	9 (8.7)	8 (30.8)	…a self-confident self-presentation.
Ambitious	4 (3.9)	4 (15.4)	…an ambitious self-presentation.
Other non-specific or rare answers	8 (7.8)	7 (26.9)	…unspecific or rare occurrences and therefore cannot be assigned to any other category.
**Influencers' view on potential improvements in self-presentation**			
Making content more interesting, personal, and aesthetic	24 (27.6)	16 (61.5)	…improving the content and relate to the quality and aesthetics of the content.
Increasing educational and informative content	13 (14.9)	7 (26.9)	…increasing the educational and informative value of the content.
Increasing authenticity	11 (12.6)	8 (30.8)	…an increase in authenticity.
Increasing the involvement of followers	9 (10.3)	8 (30.8)	…interaction with and sympathy for the followers.
Increasing activity and regularity of content	7 (8.0)	5 (19.2)	…activity on Instagram and the regularity of posts and stories.
Discarding perfectionism and showing the dark sides	7 (8.0)	5 (19.2)	…the reduction of a flawless self-presentation.
Other non-specific or rare answers	16 (18.4)	11 (42.3)	…unspecific or rare occurrences and therefore cannot be assigned to any category.
**Influencers' reasons for less authentic self-presentation**			
Criticism from followers and the private environment and fear of conflicts	16 (23.9)	8 (30.8)	…criticism and fear of conflict or criticism.
Keeping up appearances in the absence of motivation and positivity	11 (16.4)	8 (30.8)	…keeping up appearances and thus feigning a certain image of “motivation and positivity”.
Wrong cooperation partners and lack of product conviction	10 (14.9)	8 (30.8)	…the wrong cooperation partners and a lack of product conviction.
Insecurity and self-doubt	7 (10.4)	6 (23.1)	…insecurity and self-doubt.
Image editing, own perfectionism, and keeping up with ideals	7 (10.4)	5 (19.2)	…perfectionism and keeping up with ideals.
Securing privacy	4 (6.0)	4 (15.4)	…securing their privacy.
Other non-specific or rare answers	12 (17.9)	11 (42.3)	…unspecific or rare occurrences and therefore cannot be assigned to any other category.
**Influencers' reasons for using photo editing tools** ^a^			
Desire for perfection and flawlessness	21 (32.3)	15 (68.2)	... the need for beauty retouching and face filters for improved self-presentation.
Aesthetics of the pictures	16 (24.6)	13 (59.1)	…creativity, lighting conditions, and color modification.
Getting better feedback from the community	10 (15.4)	7 (31.8)	…impressing followers and getting better social feedback.
Strengthening self-confidence	7 (10.8)	7 (31.8)	…a more self-confident appearance.
Comparison and competition	5 (7.7)	5 (22.7)	…comparison processes and competition with others.
Other non-specific or rare answers	6 (9.2)	3 (13.6)	…unspecific or rare occurrences and therefore cannot be assigned to any other category.

*n*_*responses*_ represents the number of responses given by the entire sample of influencers that fell into the respective category and (in brackets) the percentage frequency in relation to all responses across all categories. *n*_*participants*_ (%) represents the total number of influencers who gave at least one answer of the corresponding category (i.e., this value represents the number adjusted for multiple answers), as well as the percentage frequency in relation to the sample size (*n* = 26). Possible missing percentages of 100 % are due to missing answers, as participants were not forced to provide responses.

^a^Twenty-two out of the 26 influencers provided answers to this question.

Second, the influencers were asked to take the perspective of their followers and assess which attributes related to self-presentation their followers would most likely ascribe to them. Interestingly, the influencers think that their followers would describe them as entertaining, positive, and motivating (69.2%), authentic, real, and approachable (65.4%), and physically attractive, stylish, and sporty (26.9%)—but also as arrogant and narcissistic (42.3%).

When asked about potential areas for improvement in their self-presentation, the influencers especially criticized their content, as they reported that it could be more interesting, personal, and aesthetic (61.5%), and more educational and informative (26.9%). Surprisingly, though perceiving their self-presentation as authentic, the influencers also see room for improvement in increasing their authenticity (30.8%) and in increasing the involvement of their followers (30.8%). These results show the BFFIs' idea of how they would describe themselves as influencers on Instagram. They play a vital role in authenticity and entertainment, which seem to be major factors of BFFIs self-presentation.

The BFFIs have also ranked their answers according to importance (for full results, see Supplementary Table 3B). 42.3% of the whole sample of influencers ranked authenticity (i.e., being authentic, real, and approachable) as the most important attribute when asked about their point of view. Further vital attributes from the influencers' point of view were confidence (26.9%) and being physically attractive, stylish, and sporty (15.4%). Similarly, when taking the perspective of their followers and thinking about what attributes they would ascribe to the BFFIs, 46.2% of the BFFIs ranked authenticity as most important characteristic of self-presentation, followed by being entertaining, positive, and motivating (23.1%), and being physically attractive, stylish, and sporty (15.4%). Surprisingly, although being arrogant and narcissistic was often mentioned as an attribute that followers supposedly ascribe to BFFIs, this attribute was not ranked as the most important category by any influencer (0.0%). With respect to potential areas for improvement in self-presentation, the BFFIs most frequently mentioned authenticity as the most crucial area for improvement (19.2%), followed by making content more interesting, personal, and aesthetic (15.4%).

In sum, influencers see their self-presentation as positive overall, regardless of whether they are asked to describe it from their own point of view or what they think how their followers perceive them. Authenticity thereby is a crucial attribute in all areas. Even though many influencers recognize that self-presentation could be associated with an arrogant and narcissistic perception by followers, the primary focus of BFFIs is to present themselves as authentic, confident, attractive, and entertaining. Nevertheless, BFFIs also still see potential for improvement in their authenticity, but also in their content (e.g., to make it more interesting, personal, aesthetic, educational and informative) and in the involvement of their followers.

#### 3.2.2 BFFIs' perceptions of their authenticity (RQ2b)

We analyzed the reported reason why the BFFIs sometimes present themselves less authentically via qualitative content analyses. The absolute and percentage frequencies of participants' answers can be found in Table 3. The three main reasons for employing a less authentic self-presentation are the BFFIs' fear of criticism from followers and the private environment and fear of conflicts (30.8% of participants gave at least one answer to this category), keeping up appearances in the absence of motivation and positivity (30.8%), and in case of wrong cooperation partners and lack of product conviction (30.8%). In addition, one-quarter of the sample (23.1%) reported insecurity and self-doubt as reasons for a less authentic self-presentation and some (19.2%) also reported image editing, own perfectionism, and keeping up with ideals.

Among the 26 influencers, 22 provided reasons for using picture editing software, which was mostly in line with the reasons for a less authentic self-presentation, for example, desire for perfection and flawlessness (68.2%) and creating aesthetics of the pictures (59.1%), getting better feedback from the community (31.8%), strengthening self-confidence (31.8%), and comparison and competition (22.7%).

So, in general, it becomes clear that own expectations and doubts as well as pressure and expectations from outside, such as followers, cooperation partners, and competition with other influencers, cause BFFIs to present themselves as (supposedly) less authentic on Instagram.

#### 3.2.3 BFFIs' tendency to respond in a socially desirable way (RQ2c)

To determine whether there was a tendency toward socially desirable behavior, we summed up the ratings of the three items of each social desirability dimension (self-deceptive enhancement and impression management) and examined whether the score reaches the value of 18, indicating socially desirable answering behavior (Paulhus, 1984). Sixteen out of 26 BFFIs (61.5%) rated their self-deceptive enhancement as lower than the threshold and 16 BFFIs (61.5%) were also below the threshold regarding impression management. So, results indicated no tendency of social desirability for the majority of BFFIs, but a third showed corresponding response tendencies.

Table 4 presents the inter-correlation matrix of all quantitative measurements, including self-deceptive enhancement and impression management. We found no significant relationships between self-deceptive enhancement and all other quantitative measurements, including impression management. In contrast, we found significant negative correlations between impression management and the influencers' attempt to appear authentic on Instagram (*r* = −0.723) as well as between impression management and the reported frequency of using picture editing tools (*r* = −0.444). There was also a significant positive correlation between impression management and the perception of ones' own role model function and associated responsibility (*r* = 0.424). In summary, the stronger the reported tendency of BFFIs for impression management, the lower their reported efforts to appear authentic, the lower the reported frequency of use of image editing programs, and the stronger their perceived own role model function and responsibility.

**Table 4:** Pearson correlations between each of the quantitative scales.

	**Importance of being an influencer**		**Celebrity brand authenticity scale**		**Attempt to appear authentic on instagram**		**Frequency of using picture editing tools**		**Compatibility of picture editing software with self-presentation**		**Social desirability: Self-deceptive enhancement**		**Social desirability: Impression management**		**Perceived role model function of influencers in general**	
	***r***	***p***	***r***	***p***	***r***	***p***	***r***	***p***	***r***	***p***	***r***	***p***	***r***	***p***	***r***	***p***
Celebrity brand authenticity scale	−0.010	0.960														
Attempt to appear authentic on Instagram	0.183	0.371	−0.408^*^	0.038												
Frequency of using picture editing tools	0.083	0.686	−0.129	0.529	0.614^***^	<0.001										
Compatibility of picture editing software with self-presentation	−0.331	0.098	−0.036	0.862	0.115	0.576	0.600^***^	0.001								
Social desirability: Self-deceptive enhancement	−0.240	0.239	0.255	0.209	−0.265	0.191	−0.139	0.499	−0.129	0.531						
Social desirability: Impression management	−0.185	0.364	0.373	0.060	−0.723^***^	<0.001	−0.444^*^	0.023	−0.126	0.541	0.054	0.794				
Perceived role model function of influencers in general	0.110	0.593	0.318	0.113	−0.055	0.788	0.224	0.271	0.115	0.575	−0.105	0.611	0.200	0.326		
Perception of one's own role model function and associated responsibility	0.046	0.822	0.343	0.086	−0.149	0.467	0.002	0.994	0.085	0.681	−0.027	0.896	0.424^*^	0.031	0.671^***^	<0.001

^*^p < 0.05, ^***^p < 0.001.

### 3.3 Acting as an influencer: how BFFIs perceive their role model function (RQ3)

#### 3.3.1 BFFIs' view on responsible and irresponsible behavior

We performed a qualitative content analysis for the open-ended questions regarding the BFFIs' view on responsible and irresponsible behavior as an influencer on Instagram (see Table 5). First, when asked to list up to five aspects that they felt constituted responsible behavior, most of the BFFIs highlighted honesty and openness toward the community (73.1% of participants provided at least one answer to this category). Further methods of acting responsibly included the wise use of their reach for educational purposes, information, and social engagement (34.6%), being aware of one's responsibility toward the community (34.6%), and the thoughtful choice of products and cooperation partners (26.9%).

**Table 5:** Categories of influencers' perceived role model function with total numbers and percentage frequencies.

**BFFIs' perceived role model function (RQ3)**			
**Topic and associated response categories**	**Total**		**Short definition of category's content**
	***n**_*responses*_* **(%)**	***n**_*participants*_* **(%)**	**All answers that refer to…**
**Influencers' view on responsible behavior as an influencer on Instagram**			
Honesty and openness toward the community	43 (47.8)	19 (73.1)	…honesty and openness toward the followers and community.
Using reach wisely for education, information, and social engagement	15 (16.7)	9 (34.6)	…the conscious use of reach for education, information, and social engagement.
Responsibility toward the community	10 (11.1)	9 (34.6)	…being conscious about the responsibility toward the community.
Thoughtful choice of products and cooperation partners	9 (10.0)	7 (26.9)	…the considered selection of products and cooperation partners
Exemplary handling of violence, drugs, and criminal behavior	5 (5.6)	4 (15.4)	…an exemplary handling of behavior that deviates from the norm and may even be punishable by law, such as violence and drugs, and observance of privacy
Open dealing with beauty retouching and beauty interventions	4 (4.4)	3 (11.5)	…an open approach to beauty retouching and beauty treatments.
Other non-specific or rare answers	4 (4.4)	3 (11.5)	…unspecific or rare occurrences and therefore cannot be assigned to any other category.
**Influencers' view on irresponsible behavior as an influencer on Instagram**			
Faking a perfect false reality and self-presentation	21 (24.4)	13 (50.0)	…a dishonest and false self-portrayal and a false image of reality.
Bullying, discrimination, and categorization	19 (22.1)	10 (38.8)	…bullying, discrimination, exclusion, and marginalization of people.
Careless and exploitative behavior toward followers	18 (20.9)	14 (53.8)	…the exploitation of followers.
Purely financial reasons and promotion of bad products	12 (14.0)	11 (42.3)	…to solely financial reasons for being an influencer and marketing of bad products.
Showing violence, drugs, pornography, and criminal behavior	8 (9.3)	6 (23.1)	…the display of violence, drugs, pornography, and other criminal behavior.
Other non-specific or rare answers	8 (9.3)	8 (30.8)	…unspecific or rare occurrences and therefore cannot be assigned to any other category.
**Influencers' ways of fulfillment of their responsibility and role model function**			
Open and honest self-presentation	24 (26.1)	18 (69.2)	…an honest and realistic self-presentation (to followers).
Aware interaction with followers and other people	15 (16.3)	12 (46.2)	…conscious approach to followers.
Increasing motivation and exemplifying a healthy lifestyle	12 (13.0)	7 (26.9)	…creating motivational content and setting an example of a healthy lifestyle.
Aware sharing of negative incidents and emotions	7 (7.6)	6 (23.1)	…to a conscious sharing of negative events and emotions with the community.
Presenting educational and informative content	7 (7.6)	7 (26.9)	…creating educational and informative content.
Aware choice of products and cooperation partners	7 (7.6)	6 (23.1)	…a conscious choice of products and cooperation partners for marketing.
Appropriate use of picture editing and filters	4 (4.3)	4 (15.4)	…the appropriate use of image editing and filters.
No depiction of violence, drugs, pornography, and criminal behavior	4 (4.3)	3 (11.5)	…refraining from showing of violence, drugs, and pornography.
Confident appearance	4 (4.3)	3 (11.5)	…a healthy mindset and self-confident self-presentation.
Other non-specific or rare answers	8 (8.7)	6 (23.1)	…unspecific or rare occurrences and therefore cannot be assigned to any other category.
**Aspects of own self-presentation with potential negative effects on followers** ^a^			
Exaggerated, always positive, and perfect self-presentation	10 (21.7)	8 (36.4)	…an exaggerated, always positive, and too perfect self-presentation.
Indifference toward followers and rash actions	10 (21.7)	6 (27.3)	…an indifference toward followers.
Being misunderstood and put in a pigeonhole	7 (15.2)	2 (9.1)	…a misunderstood perception of followers toward influencers.
Use of picture editing and filters	3 (6.5)	3 (13.6)	…photo editing and the use of filters.
No presentation of educational, informative, and helpful content	3 (6.5)	3 (13.6)	…the lack of educational, informative, and helpful content.
Marketing of products that the influencer does not support	2 (4.3)	2 (9.1)	…the trustworthiness of product marketing.
Other non-specific or rare answers	11 (23.9)	8 (36.4)	…unspecific or rare occurrences and therefore cannot be assigned to any other category.
**Influencers' implemented prevention methods regarding negative effects** ^b^			
Authentically honest and open self-presentation	14 (25.0)	14 (58.3)	…an authentic and therefore honest and open self-presentation.
Thoughtful and well-considered content	14 (25.0)	7 (29.2)	…consciously creating and sharing considered and thoughtful content.
Intentionally presenting the “dark sides” in life and negative incidents	6 (10.7)	4 (16.6)	…the depiction of the downsides of being an influencer like negative experiences.
Reducing filter use and body-related perfectionism	6 (10.7)	5 (20.8)	…discarding photo editing, filters, and perfectionism.
Involving the community	6 (10.7)	5 (20.8)	…involvement of the community.
Other non-specific or rare answers	10 (17.9)	8 (33.3)	…unspecific or rare occurrences and therefore cannot be assigned to any other category.

*n*_*responses*_ represents the number of responses given by the entire sample of influencers that fell into the respective category and (in brackets) the percentage frequency in relation to all responses across all categories. *n*_*participants*_ (%) represents the total number of influencers who gave at least one answer of the corresponding category (i.e. this value represents the number adjusted for multiple answers), as well as the percentage frequency in relation to the sample size (*n* = 26). Possible missing percentages of 100 % are due to missing answers, as participants were not forced to provide responses.

^a^Twenty-two out of the 26 influencers provided answers to this question.

^b^Twenty-four out of the 26 influencers provided answers to this question.

Second, when asked to list up to five aspects that they felt being constituted irresponsible behavior as an influencer, most BFFIs mentioned careless and exploitative behavior toward followers (53.8%) and half of the sample also criticized faking a perfect false reality and self-presentation (50%). In addition, purely financial reasons and promotion of bad products (42.3%) have been reported as irresponsible behavior by many influencers, as well as bullying, discrimination, and categorization (38.8%).

We also asked the influencers to rank their answers about responsible and irresponsible behavior according to their importance to them (Supplementary Table 3C). Honesty and openness toward the community (38.5% of the whole sample mentioned this category as first aspect), the wise use of reach for education, information, and social engagement (19.2%), and the awareness of responsibility toward the community (19.2%) were often perceived as crucial aspects of responsible behavior. Regarding irresponsible behavior, the sole pursuit of financial goals and the promotion of bad products (26.9%), careless and exploitative behavior toward followers (23.1%), and bullying, discrimination, and categorization (19.2%) were frequently viewed as the most vital aspects. In contrast, faking a perfect false reality and self-presentation was rarely seen as the most important irresponsible behavior (11.5%).

The results show that influencers focus primarily on three areas when it comes to responsible and irresponsible behavior, namely self-presentation, content characteristics, and commercialization and management of followers. This is additionally confirmed by the assessment of importance.

#### 3.3.2 BFFIs' personal handling of their role function

First, we asked the BFFIs to list up to five attributes that best describe how they fulfill their responsibility or role model function as an influencer, and conducted qualitative content analyses. Most of the BFFIs stated that they achieve this through open and honest self-presentation (69.2% of participants provided at least one answer to these categories). Other frequently mentioned methods included an aware interaction with followers and other people (46.2%), an increase of motivation and exemplifying a healthy lifestyle (26.9%), presenting educational and informative content (26.9%), and an aware choice of products and cooperation partners (23.1%).

Second, the BFFIs were asked to name up to five aspects in which they see potentially negative influences of their self-presentation on their followers. 22 out of the 26 influencers provided answers to this question and one-third of them (36.4%) stated an exaggerated, always positive, and perfect self-presentation could unfold negative consequences for followers. Moreover, several BFFIs also identified indifference toward followers and rash actions (27.3%) as problematic. Only a few influencers mentioned the usage of picture editing and filters (13.6%) and the absence of educational, informative, and helpful content (13.6%) as potentially harmful.

Third, the BFFIs were asked to name up to five aspects that they implement to prevent possible negative influences of their self-presentation on their followers. Twenty-four out of the 26 influencers provided answers to this question. BFFIs mainly reported adapting an authentically honest and open self-presentation (58.3%), creating thoughtful and well-considered content (29.2%), reducing filters and body-related perfectionism (20.8%), and involving the community (20.8%) to prevent negative effects.

In summary, like the responsible and irresponsible behavior as influencers in general, BFFIs see authentic self-presentation, the creation of valuable content, and appropriate interaction with their followers as the three key factors in being able to fulfill their role as a role model and associated responsibilities.

### 3.4 Exploratory analyses of quantitative measurements

#### 3.4.1 The role of the influencers' reach

We explored the possible impact of the number of followers on the influencers' responses. For this purpose, we calculated one-way ANOVAs with follower number (10,000–50,000 vs. 50,001–100,000 vs. >100,000 followers) as the independent variable and the nine quantitative scales listed in Table 1 as the dependent variables. For robustness checks, we re-run the analyses with two instead of three groups by combining the two sub-groups of micro-influencers and comparing them with the macro-influencers. This did not change the results, thus we only report the results of the analyses based on three groups.

We found a significant effect of follower number on the reported importance of being an influencer, *F* = 6.15, *p* = 0.007, $\eta_{\text{p}}^{2}$ = 0.348. Bonferroni-adjusted *post-hoc* tests revealed significant differences between macro-influencers with >100,000 followers on the one hand, and micro-influencers with 10,000–50,000 followers (*M*_*diff*_ = 0.93, *SE* = 0.32, *p* = 0.024) and micro-influencers with 50,001–100,000 followers (*M*_*diff*_ = 1.20, *SE* = 0.40, *p* = 0.019) on the other hand. There were no other effects of the follower number on any of the other quantitative scales, all *F*s ≤ 2.39, all *p*s ≥ 0.114, all $\eta_{\text{p}}^{2}$s ≤ 0.172.

#### 3.4.2 Correlations between quantitative measurements

We had a closer look at the inter-correlations between quantitative measurements (beyond social desirability, see Section 3.2.3). We found significant negative correlations between the Celebrity Brand Authenticity Scale and the influencers' attempt to appear authentic on Instagram (*r* = −0.408). Therefore, the higher the influencers' perception that they represent an authentic brand, the lower the reported deliberate attempt to appear authentic on Instagram.

Furthermore, we observed significant positive correlations between the attempt to appear authentic on Instagram and the frequency of using picture editing tools (*r* = 0.614), and between the frequency of using picture editing tools and the perceived compatibility of picture editing software with self-presentation (*r* = 0.600). That is, the more frequently the influencers reported using picture editing tools, the greater were their reported deliberate attempt to appear authentic and the stronger the perceived compatibility of picture editing software with an authentic self-presentation.

We also found a significant positive correlation between the perceived role model function of influencers in general and the perception of one's own role model function and associated responsibility (*r* = 0.671). This shows a high degree of congruence between the perceived social norm and one's own understanding of one's role.

## 4 Discussion

### 4.1 Main findings

The present study examined perceptions of BFFIs who actively present themselves on Instagram in Germany. We investigated their goals and motives, self-presentation, and role model function.

#### 4.1.1 Being an influencer: the goals and motives of BFFIs (RQ1)

Most of the BFFIs primarily pursue self-realization and self-development on the one hand, and economic goals, such as gaining fame and generating income through Instagram, on the other hand. The BFFIs' reported goals and motives highlight that influencers concurrently pursue intrinsic (Audrezet et al., 2020) and extrinsic goals (Fetter et al., 2023; Shabahang et al., 2022). Moreover, the overlaps in the goals they pursue for themselves and in relation to their followers also show that influencers do not differentiate strongly between them. This could be because the goals are connected in that their own goals can only be achieved through their followers. For example, fame and recognition must be achieved through self-promotion among them first to attain financial success. Influencers may therefore not be able to separate their own goals and motives from those they expect from their followers as a benchmark.

#### 4.1.2 Appearing as an influencer: BFFIs' perception of their self-presentation (RQ2)

Most influencers described their self-presentation on Instagram as authentic, entertaining, confident, and attractive. They also believe that their followers perceive them the same way, but also as narcissistic and arrogant. This indicates that influencers reflect critically on their own role, but with a willingness to take a certain amount of risk.

The BFFIs identified authenticity as the key factor for influencers' self-presentation, both as characteristic and as an area of improvement. At first glance, it therefore seems counterproductive and counterintuitive that influencers agree with adopting less authentic self-presentation and using photo editors. When looking at the reported reasons for using photo editors, though, it becomes clear that the BFFIs mainly do so because of fear of negative feedback from others, competition, keeping up appearances in the absence of motivation and positivity, wrong cooperation partners and lack of product conviction, as well as self-doubt and the need for perfection. As a result, the expectations of others and external pressure sometimes lead influencers to present themselves less authentic, even though they consider authenticity to be particularly important.

Regarding BFFIs' tendency to answer in a socially desirable way, we found that the BFFIs with an increased tendency for impression management reported fewer deliberate attempts to appear authentic, a lower frequency of using picture editing tools, and a stronger perception of their own role model function. This result may indicate a certain idea that the participants have of how a socially desirable influencer should act in front of their audience. The social ideal is that the influencer should not just attempt to appear authentic, rarely use picture editing software, and being aware of their role model function and responsibility. It is possible that these ideals could lead to a certain degree of cognitive dissonance within the influencers. Influencers use picture editing tools and have reasons for a less authentic self-presentation, though it contradicts their ideal of an influencer. This again shows that influencers care about authentic self-presentation but are not always able to implement this due to external demands and expectations.

#### 4.1.3 Acting as an influencer: how BFFIs perceive their role model function (RQ3)

The qualitative results showed that the BFFIs mainly focused on three topics about their role model function, namely authenticity, the content they produce, as well as commercialization and management of their followers.

Regarding authenticity, the BFFIs reported that they try to fulfill their role model function through an honest and open self-presentation, and with a conscious interaction with followers. In line with that, most BFFIs consider honesty and openness toward the community to be a central aspect of responsible behavior on Instagram, while faking a perfect reality and self-presentation was highlighted as irresponsible behavior with negative consequences for followers.

Regarding their content, BFFIs especially see using their reach wisely for education, information, and social engagement, and the exemplary handling of violence, drugs, pornography, and criminal behaviors as responsible behaviors.

Regarding commercialization, BFFIs perceive the sole pursuit of purely financial reasons, the promotion of bad products, and the discrimination and exploitation of followers as the most crucial aspects of irresponsible behavior. In this context, it is noteworthy that visible indicators of commercialization in the appearance of influencers can be considered indicative of fake news among young people (Zimmermann et al., 2022). As the BFFIs agreed that the commercial factor of Instagram is important to (some of) them, balancing financially successful work as an influencer while obscuring the appearance of a profit-driven agenda seems particularly challenging.

#### 4.1.4 The (minimal) impact of follower number

We found that influencers with more followers attribute greater significance and importance to being an influencer, which may reflect professionalization and the job as an influencer on Instagram as a main source of income for larger influencers. Surprisingly, there were no differences in the ratings of the remaining scales regarding self-representation and role model function, thus influencers rated these the same regardless of their size, although the requirements and expectations may change with increasing follower number (cf. Hatton, 2018).

### 4.2 Theoretical and practical implications

In addition to the detailed discussion below, a summary of the main findings and associated implications can be found in Supplementary Table 4.

#### 4.2.1 Playing the dual role of user and producer

Our findings indicate that there may be contradictions between the individual goals and motives of BFFIs. Influencers seek to realize themselves, but they also pursue financial goals that necessitate certain behaviors to make themselves attractive to potential cooperation partners, while also being a good role model for their followers. It becomes evident that influencers assume a dual role, that of an Instagram user attempting to satisfy their own needs, and that of a producer trying to meet the (sometimes opposing) needs and expectations of others. This also demonstrates the limitations of the Uses and Gratification Theory (Katz et al., 1973) in the context of influencers on social media. The theory, which has been formulated long before today's social media were born, only considers the user perspective, but not the specific situation in which the influencers find themselves: their use of Instagram does not solely depend on their own goals and motives, but also those of their followers and external partners. The theory does not cover the dual role and the interaction between the two roles. Extending the theory to include the duality of the influencers' role as users and producers and what that means for the way social media influencers use Instagram as a platform to achieve desired gratification seems a fruitful path for future research.

Balancing the diverse roles and expectations of different parties seems to be one of the most significant challenges of BFFIs, and supports and complements previous findings by ethnographic research (e.g., Davenport and Jones, 2021; Duffy and Hund, 2019). On Instagram, they take on this dual role of users and producers to achieve their own goals and motives, while they must create and maintain a desired image among their followers to gain fame. At the same time, they aim to be attractive to cooperation partners to earn money and need to prevail against other influencers as competitors. The reported reasons also indicate that BFFIs seem to have become the subject of constant evaluation on Instagram. The influencers may be exposed to immediate, intense, and easily available feedback due to the simplicity of communicating with them, even publicly, on Instagram. Indeed, followers evaluate influencers regarding characteristics like credibility, appearance, genuine self-sharing, and content creation techniques when deciding whom to follow (Tsen and Cheng, 2021). The feedback may lead to the internalization of specific ideas of how others expect influencers to present themselves. For example, our analysis of social desirability revealed the ideal picture of an influencer who not only tries to appear authentic, but also rarely uses photo editing. The BFFIs attempt to align themselves with these ideals, despite potentially adopting a less authentic self-presentation due to the expectations of others and the fear of being negatively rated. The influencers' reported reasons for adopting a less authentic self-presentation—such as fear of negative feedback from others, competition, keeping up appearances in the absence of motivation and positivity, and wrong cooperation partners and lack of product conviction—underline the need for fulfilling and balancing expectations of others. This may also result in the BFFIs' reported desire for perfection and flawlessness. It therefore seems reasonable that BFFIs are looking for a middle way between authentic and less authentic self-presentation.

The mix of authentic and less authentic self-presentation also shows the relevance of the Self-Presentation Theory (Baumeister, 1982) for the social media context in which influencer act. The theory postulates that self-presentation is orientated toward the audience's expectations if the audience is responsible for the rewards. The results of the present study now show that the self-presentation of BFFIs is based on the expectations of others, as these are essential for achieving their goals. Nevertheless, the situation of influencers on Instagram appears to be even more complex than described by the theory. Factors such as immediacy, availability, and ease of obtaining feedback, even unprompted, and the large number of different audiences with partly contradicting expectations that influencers must satisfy, seem to represent a special situation. Future research therefore can pursue an extension of the theory with these factors and examine how those factors affect self-presentation.

#### 4.2.2 Mental health issues and potential effects on followers

The pressure of balancing the various roles, the expectations of different interested parties, and the competition with other influencers also raises the question of the impact on their mental health. For instance, internalizing certain ideals, particularly those about attractiveness and competition with others have been demonstrated to be related to harmful effects on mental health (Lee-Won et al., 2020; Hoffmann and Warschburger, 2019; Kleemans et al., 2018). Combined with self-doubt and fear of criticism, this may negatively impact the influencers. These potential impacts on the BFFIs' mental health have not been studied before and may be an important focus of future research.

However, it is also important to consider potential negative consequences for the followers. Using picture editing software on social media can have a profound negative effect on the self-image of followers (Tiggemann and Zinoviev, 2019). In particular, the sexual objectification of the female body increases the risk of body dissatisfaction and eating disorders among young girls and women, influencing their behaviors (Prichard et al., 2023). The BFFI's awareness of this fact and the relative priority they ascribe to it compared to their other goals was not directly investigated in this study and can only be speculated. Nevertheless, the influencers did not mention the issue of followers' self-image in any context, although it would have been possible to acknowledge this problem. The lack of mentioning this issue indicates that the influencers either do not note it or not as much as other topics such as authenticity and financial gain.

Future research should therefore concentrate on this lack of acknowledgment in greater detail and, if necessary, incorporate educational and awareness-raising initiatives concerning the potential risks associated with idealized and inauthentic self-presentation, as well as the consequences that the use of picture editing on their followers. Initial efforts have already been made to challenge the prevailing ideals on Instagram with campaigns promoting body positivity. These have included the use of hashtag trends such as *#bodypositive* or *#nofilter*, which aim to highlight the unrealistic nature of self-presentation on the platform. However, the extent to which these hashtags resonate with their target audience and exert a lasting influence on the prevailing ideals on Instagram remains unclear. The present results indicate that the influencers note the potentially negative consequences of their self-presentation on Instagram and strive to fulfill their role model function. However, they also use picture editing tools for various reasons, including personal aspirations for flawlessness and perfectionism, and commercial considerations. Additionally, they may engage in these practices due to fear of criticism. This highlights the need for a nuanced examination of this topic. Future research could contribute to unraveling the personal barriers that hinder a desired and authentic self-presentation of influencers on Instagram to reduce the negative effects of their self-presentation.

#### 4.2.3 Appearing arrogant and narcissistic and be authentic at the same time?

The BFFIs also perceive their self-presentation rather positively in general, regardless of whether the assessment is made from their perspective or from what they believe how their followers perceive them. However, the BFFIs recognize that their followers may perceive them as arrogant and narcissistic. This circumstance raises the question of how such an impression originates. One possibility is that influencers receive corresponding feedback from followers or other users and that these comments may be salient for the influencers. Another possibility is that influencers are aware that they appear narcissistic and arrogant, which would show the BFFIs' self-critical and reflective view of their self-presentation. Alternatively, there may be an acceptance among influencers that the work and self-presentation demanded of a BFFI, such as appearing as attractive and aesthetically pleasing as possible for followers and cooperation partners, may evoke a partially arrogant and narcissistic perception among their followers. Nevertheless, influencers seem to be aware of some negative characteristics of their self-presentation. How these impressions emerge, however, can be the subject of future research.

#### 4.2.4 Realizing the role model function and social upwards comparisons

When it comes to fulfilling the influencers' role model function, one focus lies on the authenticity of the self-presentation, respectively, the presentation of a false, set-up social media persona. According to the Social Learning Theory (Bandura, 1977), people tend to imitate similar people as role models. Authenticity may foster perceived credibility and the connection between influencers and their followers (Shezala et al., 2024; Gómez, 2019). Our results now indicate that the BFFIs try to adopt an open and honest self-presentation on the one hand. On the other hand, BFFIs may be forced to present themselves as less authentic and exaggeratedly positive due to the expectations of others. Indeed, influencers were found to present themselves rather idealized (Toma, 2016). The results of the present study support this finding by revealing the BFFIs' potential reasons for such a self-presentation. These findings also highlight the issue of potential social upward comparisons, elicited among users by idealized representations on social media (Verduyn et al., 2020). According to Festinger's (1957) Social Comparison Theory, individuals use others as points of reference to assess their own abilities, opinions and behavior, particularly when those references are perceived as similar to themselves. These comparisons can be directed both downwards and upwards, i.e. comparing oneself to individuals perceived as “worse” or “better”, respectively. Such comparisons can produce both positive and negative outcomes, such as motivation to improve, but also dissatisfaction and envy (Festinger, 1957). In line with this theory, upward social comparisons were found to have positive and negative effects on followers in the context of social media influencers. Social comparisons mediate the negative relationship between Instagram use and body image dissatisfaction (Afana et al., 2021). Upward social comparisons with social media influencers also may lead to reduced wellbeing and purchase intentions (Claeys et al., 2023) and are associated with stronger feelings of envy among followers (Chae, 2017). This envy is associated with negative affective reactions, but also with positive impacts on affect and wellbeing if followers feel inspired by influencers (Lee et al., 2022; Meier and Johnson, 2022). Upwards social comparisons with influencers also seem to positively impact followers if the similarity between the influencer and the followers is highlighted (Tian et al., 2023; Kang and Liu, 2019). Thus, under specific conditions, social upward comparisons and consequently more idealized self-presentations of influencers can also positively affect followers. Therefore, the impact of influencers on their followers seems to go beyond authenticity and the assumptions of the Social Learning Theory (Bandura, 1977), which emphasizes the similarity between people. This circumstance is particularly important in relation to social media, as a balance is required between an authentic and an idealized self-presentation of the influencer.

#### 4.2.5 It is not just about self-presentation, the content counts too

Next to an authentic self-presentation, the content is another important topic of the BFFIs regarding their role model function. They reported that they try to implement thoughtful and well-considered content to prevent the negative effects of their self-presentation. They see the communication of educational, informative, and meaningful content and the responsible handling of violence, drugs and criminal behavior as part of exemplary behavior in general and their role model function and its implementation in particular. Conversely, they criticize the absence of educational, informative, and helpful content and the depiction of violence, drugs, pornography, and criminal behavior as irresponsible behavior. These findings make it clear that the BFFIs are aware of the importance of their content and its effects on followers and reflect this. This could be one of the reasons why there recently have been found positive effects (Tian et al., 2023; Lee et al., 2022; Meier and Johnson, 2022; Kang and Liu, 2019) on followers in addition to the negative effects of upward social comparisons (Claeys et al., 2023; Chae, 2017). This is supported by the circumstance that acting as a role model and motivating, inspiring, and empowering followers is the goal mentioned by most BFFIs in the present study (61.5%). Inspiration, for example, is one of the factors that mediate the impact of social media use on followers (Lee et al., 2022; Meier and Johnson, 2022). In a nutshell, good content can counteract negative effects of idealized and non-authentic self-presentation.

#### 4.2.6 The prototypical BFFI

The present study indicates that the attributes mentioned by most influencers characterize BFFIs the best from their point of view. Considering the ease of retrieval effect (Schwarz et al., 1991), it can be argued that the aspects mentioned by the influencers are particularly relevant. This is supported by the fact that the estimated importance usually corresponds to the frequency with which the attributes were mentioned. From this, a profile of the typical BFFI can be derived: the BFFIs are influencers who especially present themselves as authentic, entertaining, motivating, confident, and attractive. They strive to make their content interesting, personal, and educational. However, to reconcile the expectations of different parties, BFFIs are willing to adopt a less authentic self-presentation, if necessary, for example by using picture editing. BFFIs also take their role model function for their followers seriously, especially through an authentic and honest self-presentation, using their reach for educational and social engagement, and a thoughtful choice of marketing cooperation partners. Furthermore, they refrain from fabricating an idealized and unrealistically positive self-presentation, and they avoid personal engagement in any form of bullying and exploitative behavior toward their followers.

The development of such an influencer profile offers at least two advantages: First, it provides a template that can be used to develop such profiles for other types of influencers. This is vital because the field of influencers is very heterogeneous. Second, the profile defines certain target variables that (potential) influencers can use as a guide from a practical point of view. From a scientific point of view, such profiles enable future research to target variables and results more specifically.

### 4.3 Limitations

First, we cannot exclude the possibility of a self-selection bias as 75% of people canceled their participation in this online study, with most of them dropping out as soon as they were provided information about the survey's content on the landing page. BFFIs appear to be a hard-to-reach-group, especially when self-disclosure is required on supposedly sensitive topics. With 26 participants, the final sample is relatively small, especially regarding quantitative data and analyses. Nevertheless, the sample size is comparable to similar studies with influencers (Balaban and Szambolics, 2022; Kühn and Riesmeyer, 2021; Audrezet et al., 2020).

Second, we refrained from formulating explicit hypotheses and empirical testing and instead opted for a qualitative, exploratory approach aimed for identifying and understanding the primary topics of interest among influencers. In fact, a hypothesis-driven study would have to draw much narrower boundaries with regard to the target constructs. In addition, there is (still) a lack of established quantitative operationalisations of these constructs.

Third, the semi-open response format may have limited the topics and responses of the influencers interviewed in the present study. In completely open interviews, further or other important topics of BFFIs could have been discussed. For example, we did not ask in detail about the financial earnings of the influencers despite the high relevance of commercialization for the influencers. Indeed, before the study, we could not predict how important this topic would be for the influencers we reached. It was possible that we would primarily engage smaller influencers for whom the financial aspect may play a less prominent role. Also, addressing financial issues could have (additionally) increased dropout rate.

Fourth, the results regarding authenticity must be considered in the context of their specific operationalisation and against the background of the current zeitgeist. For example, due to the conflation of influencer as brand themselves (Gnegy, 2017), we decided to adapt the Celebrity Brand Authenticity Scale (Ilicic and Webster, 2016), which aims to capture consumer perceptions of celebrities being an authentic brand. For our study, we transformed this scale into a self-assessment. However, as Hund (2019) noted, there is no clear definition or criteria for assessing authenticity, as the criteria are constantly changing due to the dynamics of the industry. Authenticity can mean different things to different people and in different eras.

Finally, our focus was limited to BFFIs on Instagram in Germany, so the results are context specific. BFFIs may behave differently on other platforms due to different characteristics of the specific platform. It should also be noted that our findings only apply to female BFFIs. These are characterized by the need to promote their products through their (physical) self-presentation, which in turn could influence the way they present themselves. We have grouped together influencers who focus on beauty, fitness, and/or fashion because it is unclear, inter alia, where beauty ends and fashion/fitness begins. This was also shown by the fact that some participants in our study indicated covering multiple of these topics. The self-presentation of other kinds of influencers, such as those in the technology and gaming sectors, where physical appearance is not as essential to product promotion, may be completely different.

## 5 Conclusion

There is a complex interplay between personal ambitions and those of others. BFFIs have dual roles as users and producers: pursuing their personal goals and needs, being attractive and a role model for followers, asserting themselves against competitors, and winning advertising partners at the same time. They must achieve these with one social media profile, which requires the integration of sometimes contradictory processes. This challenge also seems to occasionally lead to the adoption of a less authentic, fake self-presentation, and picture editing use. This may also result in the promotion of unrealistic beauty standards and could pose a risk to the health of their followers. Based on these findings, comprehensive profile descriptions of influencers, theory-driven concretisation of constructs, and the development of urgently needed survey instruments can be derived.

## Data Availability

The raw data supporting the conclusions of this article will be made available by the authors, without undue reservation.
