# Supplementary material for: A mixed methods exploration of self-presentation, authenticity, and role model function on Instagram: perspectives from female influencers in Germany
Source: Front Psychol. 2025 Jan 22;15:1472514. doi: 10.3389/fpsyg.2024.1472514 (PMC11794495; doi:10.3389/fpsyg.2024.1472514)
Supplement: Supplementary file 1 [file Data_Sheet_1.pdf]

Supplementary Material

A mixed methods exploration of self-presentation, authenticity, and role model function on Instagram: Perspectives from female influencers in Germany

Daniel Zimmermann[1*], Colleen Schneider[1] and Kai Kaspar[1]

*** Correspondence:** Daniel Zimmermann: d.zimmermann@uni-koeln.de

Supplementary Figure 1. Questionnaire after instruction and informed consent.

Thank you very much for your honest and voluntary cooperation! We will now start with the survey.
We'll start with a few questions about your use of Instagram.

Do you describe yourself an influencer who actively and regularly presents herself on Instagram?
Single choice: "yes"; "no"

Please enter your average Instagram usage time per day in minutes.
open-ended response format

How many years have you already been active as an influencer on Instagram?
open-ended response format

Approximately how many followers do you have on Instagram?
Single choice: "10,000–50,0000"; "50,001–100,000"; ">100,000"

Which of the areas listed below best reflects your content in your feed and stories? (multiple answer options possible)
Multiple choice: "beauty"; "fitness"; "fashion"; "family"; "animals"; "food"; "travel"; "interiors"; "others (not further specified)"

Being an influencer takes great importance in my life.
five-point scale ranging from 1 ("don't agree at all") to 5 ("agree very much")

The commercial factor of Instagram is important for me.
five-point scale ranging from 1 ("don't agree at all") to 5 ("agree very much")

Please comment on the following human behaviours. To what extent does each statement apply to you personally (from 1 = "strongly disagree" to 7 = "strongly agree")?

Items from the Balanced Inventory of Desirable Responding (BIDR) by Paulhus (1984) in the short form by Winkler et al. (2006).
seven-point scale ranging from 1 ("strongly disagree") to 5 ("strongly agree")

Winkler, N., Kroh, M., & Spieß, M. (2006). *Entwicklung einer deutschen Kurzskala zur zweidimensionalen Messung von sozialer Erwünschtheit* [*Development of a German short scale for the two-dimensional measurement of social desirability*]. DIW Discussion Papers, No. 579.

I would now like to ask you about your goals as an influencer that you would like to achieve via Instagram.

List up to 5 goals that you are trying to achieve in relation to yourself with Instagram and rank these goals in order of importance (1. as the most important goal). Please keep it short. Your statement may be a maximum of 150 characters long.
open-ended response format with five text boxes, one for each answer (1. as the most important)

List up to 5 goals that you are trying to achieve in relation to your followers with Instagram and rank these goals in order of importance (1. as the most important goal). Please keep it short. Your statement may be a maximum of 150 characters long.
open-ended response format with five text boxes, one for each answer (1. as the most important)

In the following, I would like to ask you a few questions about your self-presentation on Instagram.
List up to 5 attributes (characteristics, features, qualities) that best describe how you present yourself on Instagram and rank them in order of importance (1. as the most important attribute). Your statement may be a maximum of 150 characters long.

open-ended response format with five text boxes, one for each answer (1. as the most important)

Now take the perspective of your followers. Which attributes (characteristics, features, qualities) of your self-presentation would your followers most likely ascribe to you? Rank these attributes in order of importance (1. as the most important attribute). Your statement may be a maximum of 150 characters long.

open-ended response format with five text boxes, one for each answer (1. as the most important)

List up to 5 aspects where you personally see potential for improvement in your self-presentation on Instagram and rank them in order of importance (1. as the biggest weakness). Your statement may be a maximum of 150 characters long.

open-ended response format with five text boxes, one for each answer (1. as the most important)

Please indicate how much you agree with the following statements about your self-presentation on Instagram (from 1 = "don't agree at all" to 5 = "agree very much").

It does matter to me how I appear to my followers and how they perceive me.

five-point scale ranging from 1 ("don't agree at all") to 5 ("agree very much")

I try to behave on Instagram in a manner that is consistent with my held values, even if others criticize or reject me for doing so.

five-point scale ranging from 1 ("don't agree at all") to 5 ("agree very much")

As an influencer on Instagram, I care about openness and honesty.

five-point scale ranging from 1 ("don't agree at all") to 5 ("agree very much")

As an influencer on Instagram, I place a good deal of importance on others understanding who I really am.

five-point scale ranging from 1 ("don't agree at all") to 5 ("agree very much")

My followers can count on me being who I am regardless of the situation.

five-point scale ranging from 1 ("don't agree at all") to 5 ("agree very much")

I am very authentic on Instagram and can simply be myself.

five-point scale ranging from 1 ("don't agree at all") to 5 ("agree very much")

As an influencer, I sometimes just try to 'appear authentic'.

five-point scale ranging from 1 ("don't agree at all") to 5 ("agree very much")

List up to 5 reasons why you may have presented yourself as less authentic or less "as yourself" to your followers. Your statement may be a maximum of 150 characters long.

open-ended response format with five text boxes, one for each answer

How often do you use photo editors, face filters, and other similar tools for your stories and posts on Instagram?

five-point scale ranging from 1 ("not at all") to 5 ("always")

If you use image editing programs: List up to 5 reasons why <u>you use image editing programs, face filters & the like for your stories and posts on Instagram</u>. Your statement may be a maximum of 150 characters long.

open-ended response format with five text boxes, one for each answer

The use of face filters and beauty retouching is compatible with my intended goals of my self-presentation on Instagram.

five-point scale ranging from 1 ("don't agree at all") to 5 ("agree very much")

The use of face filters and beauty retouching is compatible with authentic self-presentation.

five-point scale ranging from 1 ("don't agree at all") to 5 ("agree very much")

Finally, I have a few general questions about the role model function of influencers. Please indicate how much you agree with the following statements (from 1 = "don't agree at all" to 5 = "agree very much").

Influencers generally have a role model function for their followers on Instagram.

five-point scale ranging from 1 ("don't agree at all") to 5 ("agree very much")

Influencers are important as role models for their followers.

five-point scale ranging from 1 ("don't agree at all") to 5 ("agree very much")

List up to 5 aspects that you consider to be <u>responsible</u> behavior as an influencer on Instagram and rank them in order of importance (1. as the most important aspect). Your statement may be a maximum of 150 characters long.

open-ended response format with five text boxes, one for each answer (1. as the most important)

List up to 5 aspects that you consider to be <u>irresponsible</u> behavior as an influencer on Instagram and rank them in order of importance (1. as the most important aspect). Your statement may be a maximum of 150 characters long.

open-ended response format with five text boxes, one for each answer (1. as the most important)

Please indicate how much you agree with the following statements <u>about your own responsibility towards your followers</u> (from 1 = "don't agree at all" to 5 = "agree very much").

I am aware of a certain responsibility towards my followers on Instagram.

five-point scale ranging from 1 ("don't agree at all") to 5 ("agree very much")

I am aware of my influence as an influencer on my followers.

five-point scale ranging from 1 ("don't agree at all") to 5 ("agree very much")

I know that I may also negatively influence my followers through falsely conveying ideals of my self-presentation on Instagram.

five-point scale ranging from 1 ("don't agree at all") to 5 ("agree very much")

In my opinion, my followers strongly orientate to me and my posts.

five-point scale ranging from 1 ("don't agree at all") to 5 ("agree very much")

About my self-presentation as an influencer, I demonstrate responsible handling of my role model function towards my followers.

five-point scale ranging from 1 ("don't agree at all") to 5 ("agree very much")

List up to 5 attributes (characteristics, features, qualities) of your self-presentation, showing how you fulfill your responsibility or role model function as an influencer. Your entry may be a maximum of 150 characters long.

open-ended response format with five text boxes, one for each answer

List up to 5 aspects where you see negative influences of your self-presentation regarding your role model function for your followers or where you do less justice to this role. Your entry may be a maximum of 150 characters long.

open-ended response format with five text boxes, one for each answer

List up to 5 aspects that you implement to prevent possible negative influences of your self-presentation on Instagram towards your followers. Your entry may be a maximum of 150 characters long.

open-ended response format with five text boxes, one for each answer

Thank you very much for your participation!
You can now close the page.

Supplementary Table 1. Results of the exploratory factor analysis (varimax rotation) for self-developed scales.

	Factor loadings
**BFFIs' goals and motives (RQ1)**	
Importance of being an influencer (Cronbach's α = 0.780)	
Being an influencer takes great importance in my life	0.906
The commercial factor of Instagram is important for me	0.906
**BFFIs' perception of their self-presentation, authenticity, and tendency to respond in a socially desirable way (RQ2a – RQ2c)**	
Attempt to appear authentic on Instagram (Cronbach's α = 0.701)[1]	
It does matter to me how I appear to my followers and how they perceive me	0.884
I am very authentic on Instagram and can simply be myself	–0.803
As an influencer, I sometimes just try to 'appear authentic'	0.682
Compatibility of picture editing software with self-presentation (Cronbach's α = 0.895)	
The use of face filters & beauty retouching is compatible with my intended goals of my self-presentation on Instagram[1]	0.951
The use of face filters and beauty retouching is compatible with authentic self-presentation	0.951
**BFFIs' perception of their role model function (RQ3)**	
Perceived role model function of influencers in general (Cronbach's α = 0.896)	
Influencers generally have a role model function for their followers on Instagram	0.952
Influencers are important as role models for their followers	0.952
Perception of one's own role model function and associated responsibility (Cronbach's α = 0.824)	
I am aware of a certain responsibility towards my followers on Instagram	0.816
I am aware of my influence as an influencer on my followers	0.777
I know that I may also negatively influence my followers through falsely conveying ideals of my self-presentation on Instagram	0.763
In my opinion, my followers strongly orientate to me and my posts	0.760
About my self-presentation as an influencer, I demonstrate responsible handling of my role model function towards my followers	0.749

Note. The factor analyses supported the intended single-factorial structure of each scale, with all Kaiser-Meyer-Olkin measures of sampling adequacy > 0.50 and all Bartlett's tests of Sphericity being significant ($p \leq 0.001$).

[1] The exploratory factor analysis revealed a negative loading of the second item on the factor. This was interpreted to suggest that the first and third items of the scale are about trying to make an authentic impression on others. The second item is about actually being authentic. This results in the opposite loading of the second item. Therefore, in the following analysis, the second item was negatively poled and recoded. Higher values indicate a stronger attempt to appear authentic to the followers.

Supplementary Table 2a. Sample answers for each category of responses regarding influencers' goals and motives.

BFFIs' goals and motives (RQ1)	
**Topic and associated response categories**	**Sample Answers**
Goals and motives that influencers pursue for themselves	
Self-realisation and self-development	Get to know myself better; self-confidence
Self-promotion and increasing public awareness level	Marketing myself; More active followers
Serving and acting as a role model and motivating, inspiring, and empowering followers	Provide people with motivation and help; give them self-confidence
Making money	Earn money with Instagram
Being inspired, motivated, informed, and entertained	I want to be inspired by others; motivation for myself
Establishing contact to and bidirectional exchange with others	Exchange with other people
Spreading joy and fun	Bringing joy to others; entertaining people
Presenting educational and informative content	Developing creative new content; giving people added value
Other non-specific or rare answers	Honesty; Instagram is much more approachable than the press
Goals and motives that influencers pursue in relation to their followers	
Serving and acting as a role model and motivating, inspiring, and empowering followers	Motivate people; provide a platform for a positive mindset
Establishing contact and bidirectional exchange with others	Getting to know new people; building a community that suits me as a person and enriches me.
Self-promotion and increasing public awareness level	Get more followers and story views to get better paid placements; Perfect advertising platform for my content
Presenting educational and informative content	I want to create added value for my followers; content that proves to be helpful
Being perceived as sympathetic, authentic, and honest	Being real; showing people my life honestly; presenting real life
Getting appreciation and acknowledgement	I want to gain trust and be perceived as a likeable person; admiration
Communicating an own point of view on a topic	
Self-realisation and development	To learn to be critical; learn from followers
Other non-specific or rare answers	No fear of socially inappropriate topics

Supplementary Table 2b. Sample answers for each category of responses regarding influencers' perceived self-presentation.

**BFFIs' perceived self-presentation (RQ2a, RQ2b)**	
**Topic and associated response categories**	**Sample Answers**
Influencers' description of own self-presentation	
Authentic, real, and approachable	Authentic; realistic; honest
Entertaining, positive, and motivating	Motivating; sympathetic; kind; happy
Confident	Self-confident appearance; confident
Physically attractive, stylish, and sporty	Sporty; attractive; sexy;
Ambitious	Someone who never gives up; disciplined
Perfectionistic	Perfect life; perfectionist
Other non-specific or rare answers	Unapproachable; emotional
Influencers' beliefs of followers' perception of own self-presentation	
Entertaining, positive, and motivating	Humorous; motivating; cheerful
Authentic, real, and approachable	Approachable; to be genuine; honest
Arrogant and narcissistic	Closed off/very private in some situations; perhaps a little arrogant
Physically attractive, stylish, and sporty	Stylish; sporty
Confident	Self-confident appearance; self-confident
Ambitious	Who follow you more closely (stories) as super motivated and determined
Other non-specific or rare answers	Quick-tempered; smart
Influencers' view on potential improvements in self-presentation	
Making content more interesting, personal, and aesthetic	Entertainment factor. I find it difficult to come out of my shell and convey my genuine humor/my genuine nature.
Increasing educational and informative content	To do more in terms of expressing opinions; a little less self-promotion, but more content that is helpful
Increasing authenticity	Openness; Re-recording of stories (is not so real)
Increasing the involvement of followers	More interaction; Be more active with the community, respond more to messages
Increasing activity and regularity of content	More content; more accounts
Discarding perfectionism and showing the dark sides	Not being so perfectionist; my biggest weakness is not showing the dark side
Other non-specific or rare answers	Personality; Keeping motivation at work permanently high
Influencers' reasons for less authentic self-presentation	
Criticism from followers and the private environment and fear of conflicts	Lack of understanding; fear of criticism
Keeping up appearance in the absence of motivation and positivity	If you are in a bad mood and still have to do a story (for advertising or self-promotion)
Wrong cooperation partners and lack of product conviction	Selling a product; long-term advertising contracts. Sometimes it happens that you like a brand overall, but not a new product, for example.
Insecurity and self-doubt	Shame; I also sometimes find it difficult to come out of myself in front of strangers in real life.
Image editing, own perfectionism, and keeping up with ideals	Putting up a better image of myself; you want pictures as great as other influencers
Securing privacy	Keep private's as private; Private incidents
Other non-specific or rare answers	Achieving a goal; it's important to me not to have to pretend anything
Influencers' reasons for using photo editing tools	
Desire for perfection and flawlessness	Discreet concealment of unfavourable problem areas of the skin; to look beautiful and attractive
Aesthetics of the pictures	You can often "save" a picture with filters; it's art
Getting better feedback from the community	Achieve added value in followers; such postings get more likes
Strengthening self-confidence	You feel better and prettier; self-confidence and recognition
Comparison and competition	Everyone uses filters; comparison with other influencers
Other non-specific or rare answers	You can continue to develop; lips and nose remain my own

Supplementary Table 2c. Sample answers for each category of responses regarding Influencers' perceived role model function.

BFFIs' perceived role model function (RQ3)	
**Topic and associated response categories**	**Sample Answers**
Influencers' view on responsible behaviour as an influencer on Instagram	
Honesty and openness towards the community	Admitting mistakes; being able to show your flaws; honesty and not lying
Using reach wisely for education, information, and social engagement	Education; using his voice for important topics and informing his followers about important topics.
Responsibility towards the community	Appreciation of followers; responsible interaction
Thoughtful choice of products and cooperation partners	Stand for what you advertise; market products not just for the money
Exemplary handling of violence, drugs, and criminal behaviour	No glorification of drugs; violence is not glorified
Open dealing with beauty retouching and beauty interventions	Talk openly about image processing, communicate methods clearly
Other non-specific or rare answers	Gain confidence; Love yourself; Safe place
Influencers' view on irresponsible behaviour as an influencer on Instagram	
Faking a perfect false reality and self-presentation	Showing off your prolly life; false self-promotion; lying with story content, products, and images
Bullying, discrimination, and categorisation	Spreading hate; bullying; judging people you don't know
Careless and exploitative behaviour towards followers	Wrong sources; communicating bad values; borderline statements
Purely financial reasons and promotion of bad products	Promote products/services that you don't stand behind; only present collaborations for money
Showing violence, drugs, pornography, and criminal behaviour	Taking drugs in a live stream or similar; incitement to violence
Other non-specific or rare answers	To compare; own conviction
Influencers' ways of fulfilment of their responsibility and role model function	
Open and honest self-presentation	I am honest; I am transparent and talk about my own development/experience
Aware interaction with followers and other people	Convey a sense of belonging (no exclusion); respond to criticism
Increasing motivation and exemplifying a healthy lifestyle	Sporty lifestyle; mental training
Aware sharing of negative incidents and emotions	I also show sad moments (rarely); I also show myself on bad days when I'm not perfectly styled
Presenting educational and informative content	I share information about topics that I consider socially relevant (e.g. social, political, world events)
Aware choice of products and cooperation partners	Only cooperation of products I like; I choose my cooperation partners carefully.
Appropriate use of picture editing and filters	Show myself without filters and beauty retouching; I talk openly about image editing and posing tricks
No depiction of violence, drugs, pornography, and criminal behaviour	I try not to show alcohol and cigarettes on my profile
Confident appearance	Strong self-confident appearance; Having an own opinion
Other non-specific or rare answers	Don't get too negative either; be self-confident; always work on myself
Aspects of own self-presentation with potential negative effects on followers	
Exaggerated, always positive, and perfect self-presentation	I show a lot of skin and perfect pictures; always positive representation of myself
Indifference towards followers and rash actions	Rash actions; violence; disrespect
Being misunderstood and put in a pigeonhole	You are pigeonholed; you can be misunderstood
Use of picture editing and filters	I often use filters; constantly using filters and editing images
No presentation of educational, informative, and helpful content	Drawing too little attention to important topics/donations, leading by example on certain topics
Marketing of products that the influencer does not support	Advertising for things you are not 100% behind
Other non-specific or rare answers	Loud; recognizing reluctance
Influencers' implemented prevention methods regarding negative effects	
Authentically honest and open self-presentation	I always try to be myself; always communicate openly about everything
Thoughtful and well-considered content	Avoiding drugs; researching sources; addressing issues such as excessive behaviour
Intentionally presenting the "dark sides" in life and negative incidents	Don't always show yourself in top form, we all have quirks; if you want me to get fatter, just show that too
Reducing filter use and body-related perfectionism	Post photos that aren't perfect; Don't use filters; I don't edit my body in pictures.
Involving the community	Communication; I answer some of my followers' questions so that they don't think I always look so perfect
Other non-specific or rare answers	I behave in accordance with the general values; I only work when I feel like it and am in a good mood

Supplementary Table 3a. Percentage frequency of influencers (n = 26) who ranked goals and motives of the respective category at the corresponding position in the importance hierarchy (with 1. as the most important category).

BFFIs' goals and motives (RQ1)					
	1. %	2. %	3. %	4. %	5. %
Goals and motives that influencers pursue for themselves					
Self-realisation and self-development	23.1	15.4	15.4	19.2	11.5
Self-promotion and increasing public awareness level	15.4	26.9	19.2	7.7	0.0
Serving and acting as a role model and motivating, inspiring, and empowering followers	15.4	15.4	7.7	7.7	7.7
Making money	19.2	3.8	11.5	3.8	11.5
Being inspired, motivated, informed, and entertained	7.7	15.4	7.7	7.7	7.7
Establishing contact to and bidirectional exchange with others	7.7	3.8	0.0	3.8	15.4
Spreading joy and fun	3.8	3.8	11.5	0.0	0.0
Presenting educational and informative content	0.0	0.0	3.8	11.5	0.0
Other non-specific or rare answers	7.7	11.5	15.4	7.7	3.8
Goals and motives that influencers pursue in relation to their followers					
Serving and acting as a role model and motivating, inspiring, and empowering followers	42.3	11.5	38.5	11.5	15.4
Establishing contact and bidirectional exchange with others	3.8	19.2	15.4	23.1	7.7
Self-promotion and increasing public awareness level	19.2	11.5	3.8	7.7	7.7
Presenting educational and informative content	19.2	23.1	3.8	0.0	3.8
Being perceived as sympathetic, authentic, and honest	7.7	15.4	3.8	3.8	0.0
Getting appreciation and acknowledgement	0.0	7.7	0.0	11.5	7.7
Communicating an own point of view on a topic	3.8	3.8	3.8	3.8	3.8
Self-realisation and development	0.0	7.7	7.7	0.0	0.0
Other non-specific or rare answers	3.8	0.0	0.0	3.8	7.7

Note. The individual values per ranking place do not necessarily add up to 100%, as the participants were not obliged to give five answers.

Supplementary Table 3b. Percentage frequency of influencers (*n* = 26) who ranked self-presentation aspects of the respective category at the corresponding position in the importance hierarchy (with 1. as the most important category).

**BFFIs' perceived self-presentation (RQ2a)**					
	1. %	2. %	3. %	4. %	5. %
Influencers' description of own self-presentation					
Authentic, real, and approachable	42.3	19.2	23.1	34.6	11.5
Entertaining, positive, and motivating	11.5	30.8	53.8	7.7	7.7
Confident	26.9	11.5	3.8	7.7	11.5
Physically attractive, stylish, and sporty	15.4	7.7	3.8	7.7	7.7
Ambitious	0.0	11.5	3.8	3.8	7.7
Perfectionistic	0.0	7.7	0.0	3.8	0.0
Other non-specific or rare answers	3.8	11.5	7.7	3.8	11.5
Influencers' beliefs of followers' perception of own self-presentation					
Entertaining, positive, and motivating	23.1	30.8	30.8	15.4	15.4
Authentic, real, and approachable	46.2	11.5	23.1	15.4	11.5
Arrogant and narcissistic	0.0	19.2	11.5	11.5	11.5
Physically attractive, stylish, and sporty	15.4	15.4	3.8	3.8	0.0
Confident	11.5	11.5	7.7	3.8	0.0
Ambitious	3.8	0.0	3.8	0.0	7.7
Other non-specific or rare answers	0.0	11.5	7.7	7.7	3.8
Influencers' view on potential improvements in self-presentation					
Making content more interesting, personal, and aesthetic	15.4	38.5	11.5	11.5	15.4
Increasing educational and informative content	11.5	15.4	11.5	7.7	3.8
Increasing authenticity	19.2	11.5	7.7	3.8	0.0
Increasing the involvement of followers	11.5	0.0	11.5	11.5	0.0
Increasing activity and regularity of content	3.8	3.8	7.7	3.8	7.7
Discarding perfectionism and showing the dark sides	11.5	7.7	7.7	0.9	0.0
Other non-specific or rare answers	26.9	11.5	15.4	3.8	3.8

Note. The individual values per ranking place do not necessarily add up to 100%, as the participants were not obliged to give five answers.

Supplementary Table 3c. Percentage frequency of influencers (*n* = 26) who ranked responsible and irresponsible behaviour of the respective category at the corresponding position in the importance hierarchy (with 1. as the most important category).

**BFFIs' role model function (RQ3)**					
	1.<br>%	2.<br>%	3.<br>%	4.<br>%	5.<br>%
Influencers' view on responsible behaviour as an influencer on Instagram					
Honesty and openness towards the community	38.5	53.8	30.8	26.9	15.4
Using reach wisely for education, information, and social engagement	19.2	11.5	15.4	7.7	3.8
Responsibility towards the community	19.2	7.7	3.8	3.8	3.8
Thoughtful choice of products and cooperation partners	15.4	11.5	7.7	0.0	0.0
Exemplary handling of violence, drugs, and criminal behaviour	3.8	7.7	7.7	0.0	0.0
Open dealing with beauty retouching and beauty interventions	0.0	3.8	7.7	3.8	0.0
Other non-specific or rare answers	3.8	0.0	0.0	7.7	3.8
Influencers' view on irresponsible behaviour as an influencer on Instagram					
Faking a perfect false reality and self-presentation	11.5	30.8	19.2	15.4	3.8
Bullying, discrimination, and categorisation	19.2	15.4	19.2	11.5	7.7
Careless and exploitative behaviour towards followers	23.1	11.5	11.5	15.4	7.7
Purely financial reasons and promotion of bad products	26.9	15.4	0.0	3.8	0.0
Showing violence, drugs, pornography, and criminal behaviour	11.5	11.5	3.8	0.0	3.8
Other non-specific or rare answers	7.7	7.7	11.5	3.8	0.0

Note. The individual values per ranking place do not necessarily add up to 100%, as the participants were not obliged to give five answers.

Supplementary Table 4. Summary of main findings and their theoretical and practical implications.

Main findings	Theoretical and practical Implications
**Being an influencer: What goals and motives do BFFIs pursue? (RQ1)**	
a) Self-realisation, being a responsible role model, and economic goals as the most common and important goals influencers pursue for themselves and in relation to their followers.	1) Illustrates the influencers' dual role as users themselves who pursue their own goals and motives and producers who make content for their followers and cooperation partners.<br>2) The Uses and Gratification Theory (Katz et al., 1973) only covers the user perspective and thus shows limitations in its application in the context of social media influencers.<br>3) Potential need for influencers to balance their own needs with those of their followers and cooperation partners, while competing with other influencers. This tension between goals and demands impact the influencers' self-presentation, forcing them to sometimes not present themselves as authentically as they would like. This pressure and potential criticism by followers because of commercial goals and inauthenticity (Zimmermann et al., 2022) may also result in (psychological) harm for the influencers.
**Appearing as an influencer: How do BFFIs perceive their own self-presentation? (RQ2)**	
a) BFFI perceive their self-presentation mainly as authentic, confident, and entertaining, and they believe that their followers perceive them this way, too. Though, use of picture editing and filters are perceived as compatible with authentic self-presentation and influencers' motives.<br>b) Some influencers believe that their followers perceive them as arrogant and narcissistic, nevertheless.<br>c) Increasing authenticity of their self-presentation as the biggest area for improvement reported by BFFI.<br>d) Fear of criticism, keeping up appearances in the absence of motivation and positivity, competition, and insecurity as main reasons for a non-authentic self-presentation.<br>e) Increasing impression management tendencies are connected to reduced attempts to appear authentic and reduced frequency of using picture editing tools.	1) Influencers must balance between an authentic and a posed self-presentation to fulfil the requirements of different parties (followers and cooperation partners) while also competing with other influencers. This may be the reason why BFFIs seem to be convinced they present themselves authentically and generally positive though they also report adopting inauthentic self-presentation from time to time (for example by using picture editing programs).<br>2) On the one hand, the findings about trying to appear authentic and the reasons for a less authentic self-presentation are according to the Self-Presentation Theory (Baumeister, 1982) which postulates that self-presentation orients towards audiences if the audience is responsible for the rewards. On the other hand, the theory does not consider the immediacy and scale of the potential feedback and criticism influencers may be exposed, and the number of different interested parties / audiences that influencers may have to please, which also may have contradicting expectations.<br>3) Influencers did not mention that using picture editing software on social media can have a profound negative effect on the self-image of followers (Tiggemann & Zinoviev, 2019). Thus, influencers either do not recognise it or not as much as other topics. Influencers may promote awareness through campaigns.<br>4) Influencers may also recognise that the work and self-presentation demanded of a BFFI, such as appearing as attractive, may evoke a partially arrogant and narcissistic perception among their followers.
**Acting as an influencer: How do BFFIs perceive their role model function? (RQ3)**	
a) Honesty and openness towards the community, wise use of reach for education, information, and social engagement, as well as being aware of one's responsibility towards the community were reported as the main aspects of responsible behaviour.<br>b) Careless and exploitative behaviour, faking a perfect reality and self-presentation, as well as purely financial reasons and promotion of bad products were reported as main aspects of irresponsible behaviours.<br>c) BFFIs reported to be aware of their role model function and to realise it primarily by an authentic and honest self-presentation.<br>d) An exaggerated and perfect self-presentation was most frequently identified by BFFIs as area with potential negative effects on their followers.<br>e) BFFI try to prevent negative effects by creating considered and thoughtful content.	1) BFFIs do recognize their role model function and have a certain image and idea of what defines responsible and irresponsible behaviour on Instagram. The focus thereby lies on authenticity of the self-presentation, their content, and commercialisation and management of their followers. Due to the need of fulfilling the expectations of different parties, a discrepancy may be found between the desire to be a role model and the actual self-presentation.<br>2) Though influencers value authenticity and criticise a false image to their followers, they present themselves unauthentic and positive to keep up a certain image for followers and cooperation partners, fearing negative feedback. The resulting idealised self-presentation of influencers may elicit upwards social comparisons and thus have a potentially harmful effects on followers. Influencers may counteract negative effects on followers with their produced content.<br>3) According to the Social Learning Theory (Bandura, 1977), people tend to imitate especially individuals they perceive as similar and authentic. The (positive) effect of upward social comparisons on followers' attitudes and behaviours suggests that social learning in the context of social media influencers may be more complex, though.
